# In Silico Investigation of Phytochemicals From Clinically Tested Herbal Extracts as Potential Dihydrofolate Reductase Inhibitors for Buruli Ulcer

**DOI:** 10.1155/bmri/4196295

**Published:** 2025-09-03

**Authors:** Alkhair Adam Khalil Mohamed, Tamekloe Woasiedem, Philip Collins Appiah-Ofori, Esraa Mohammed Abdulmahmoud Adam, Samuel Asiamah Obiri, Lawrence Sheringham Borquaye, Christian K. Adokoh, Ghazi Elamin, Isaac Asiamah

**Affiliations:** ^1^ Department of Biomedical Sciences, School of Allied Health Sciences, College of Health and Allied Sciences, University of Cape Coast, Cape Coast, Ghana, ucc.edu.gh; ^2^ Department of Chemistry, School of Physical Sciences, College of Agriculture and Natural Sciences, University of Cape Coast, Cape Coast, Ghana, ucc.edu.gh; ^3^ Department of Chemistry, Kwame Nkrumah University of Science and Technology, Kumasi, Ghana, knust.edu.gh; ^4^ Central Laboratory, Kwame Nkrumah University of Science and Technology, Kumasi, Ghana, knust.edu.gh; ^5^ Department of Forensic Sciences, School of Biological Sciences, College of Agriculture and Natural Sciences University of Cape Coast, Cape Coast, Ghana; ^6^ Department of Pharmaceutical Chemistry, College of Pharmacy, Karary University, Khartoum, Sudan, karary.edu.sd

**Keywords:** Buruli ulcer, dihydrofolate reductase (DHFR), *in silico* drug discovery, *Mangifera indica*, MD simulations, molecular docking, *Mycobacterium ulcerans*, *Nicotiana tabacum*, phytochemicals

## Abstract

Buruli ulcer (BU), a neglected tropical disease caused by *Mycobacterium ulcerans*, requires more effective and accessible treatments. Clinical trials have shown the efficacy of herbal formulations containing *Nicotiana tabacum*, *Mangifera indica*, *Carica papaya*, and *Solanum rugosum*, although the active phytochemicals remain unstudied. In the present study, we employed an *in silico* approach to identify the active phytochemicals from these plants that could inhibit *M. ulcerans* dihydrofolate reductase (*Mu*DHFR), a crucial enzyme for mycobacterial survival. A library of 172 phytochemicals was screened using molecular docking, followed by 300‐ns molecular dynamics (MD) simulations using AMBER for the top hits and standard drugs (methotrexate and trimethoprim). Binding free energies were calculated using the molecular mechanics/generalized born surface area (MM/GBSA) method. The extended MD simulations and post hoc MM/GBSA analysis revealed that kaempferol‐3‐O‐rutinoside (*Δ* *G*_bind_ −51.62 kcal/mol) and neochlorogenic acid (*Δ*G_bind_ −49.44 kcal/mol) exhibited the strongest and most stable binding to *Mu*DHFR. The binding energies were more favorable than both methotrexate (*Δ* *G*_bind_ −44.18 kcal/mol) and trimethoprim (*Δ* *G*_bind_ −41.01 kcal/mol) used as positive controls. Stability analyses (RMSD and RMSF) and principal component analysis (PCA) confirmed that these compounds form stable complexes and induce specific conformational states in the enzyme. Favorable ADMET profiles, particularly the lack of predicted skin sensitization, support their potential for topical use. This study identifies kaempferol‐3‐O‐rutinoside and neochlorogenic acid as the most promising lead candidates for developing novel BU therapies, warranting their prioritization for experimental validation.

## 1. Introduction

Buruli ulcer (BU), a neglected tropical disease caused by *Mycobacterium ulcerans*, leads to devastating skin and soft tissue destruction [1]. In 2023, out of 1952 suspected cases of BU from 12 countries, 1573 came from countries in Africa [2]. Although the current WHO‐recommended antibiotic therapy is effective, limitations including cost, the need for injections, and toxicity continue to hinder accessibility, particularly in remote endemic areas [3]. Consequently, there is an urgent need for more accessible and affordable therapeutic alternatives. African traditional herbal medicines offer a promising avenue, especially given their widespread use and reported efficacy in resource‐limited settings [4]. A clinical trial study conducted in the Ivory Coast with a topical herbal formulation comprising *Nicotiana tabacum*, *Mangifera indica*, *Carica papaya*, and *Solanum rugosum* demonstrated an 80% healing rate [5, 6]. Despite this compelling clinical outcome, the specific bioactive phytochemicals responsible for the therapeutic effect and their molecular targets within *M. ulcerans* remain unknown. Identifying these active components and their molecular targets is expected to aid drug discovery efforts to identify and optimize lead compounds for further development as therapeutic agents against BU [7].

Dihydrofolate reductase (DHFR) is an essential enzyme in folate metabolism, critical for DNA synthesis and mycobacterial survival, making it a validated drug target [8–11]. While antifolate drugs like trimethoprim and methotrexate are known, they exhibit poor activity against mycobacterial DHFRs due to permeability issues or intrinsic resistance [9]. Nevertheless, previous studies have demonstrated the antifolate activity of several classes of phytochemicals against DHFR of *Mycobacterium tuberculosis* [12], *Staphylococcus aureus* [13], *Homo sapiens* [14, 15], and *Plasmodium falciparum* [16]. This highlights the potential for discovering natural product‐based novel inhibitors effective against *M. ulcerans* DHFR (*Mu*DHFR).

This study employed an *in silico* approach to elucidate the specific phytochemicals within the clinically proven herbal formulation against *M. ulcerans*. We hypothesize that the efficacy of the herbal remedy stems from phytochemicals inhibiting *Mu*DHFR. To test this, we constructed a library of 172 phytochemicals previously identified from the four plants [7]. Using integrated computational methods—molecular docking to screen for potential binders, Prime MM‐GBSA for initial binding affinity ranking, followed by extensive 300‐ns molecular dynamics (MD) simulations with MM/GBSA binding free energy calculations, and ADMET profiling—we systematically identified and evaluated high‐affinity candidate inhibitors. The novelty of this work lies in its focus on phytochemicals derived from a formulation with demonstrated clinical efficacy against BU, employing advanced simulations to provide a dynamic assessment of binding stability and affinity. This integrated strategy is aimed at pinpointing the most promising natural product leads for developing novel, accessible BU therapies targeting *Mu*DHFR.

## 2. Materials and Methods

### 2.1. Ligand Preparation

A library of 172 phytochemicals was built from compounds that have been previously elucidated from the four medicinal plants (*N. tabacum, M. indica, S. rugosum*, and *C. papaya*). The compounds were selected following an extensive literature review of phytochemical studies on these plants [7]. This approach was intentionally adopted to ensure an unbiased initial screening, free from prior assumptions about biological activity. The full list of the phytochemicals and their sources is presented in the Table S1. The SMILES of the 172 phytochemicals and two standard DHFR inhibitors, methotrexate and trimethoprim, were retrieved from the PubChem database and imported to the Maestro software in Schrödinger suite [17]. The LigPrep tool [18] was used to convert the SMILES into 3D structures and then to conduct energy minimization under the OPLS4 force field [19] at the default settings. LigPrep was also used to desalt and generate possible ionization states for each ligand at pH = 7.0 ± 2. The final library of prepared phytochemicals and standard drugs was used to perform the molecular docking study.

### 2.2. Protein Preparation

The crystallized structure of *M. ulcerans* dihydrofolate reductase (*Mu*DHFR) was obtained as a pdb file from the protein data bank, accessible at https://www.rcsb.org/structure/6UWW under the identification code (PDB ID: 6UWW). The structure was solved by X‐ray diffraction at a resolution of 0.92 Å, with a free *R* value of 0.145, a work *R* value of 0.132, and an observed *R* value of 0.132. The pdb file of the target enzyme was loaded to the protein preparation workflow in Maestro at the default settings. The target enzyme was prepared through a series of sequential steps, involving filling in missing side chains, assigning bond order, replacing hydrogens, generating het states at pH 7.4 ± 2, deleting bulk waters, optimizing hydrogen‐bond assignments at pH 7.4, and energy minimization using the OPLS4 force field.

### 2.3. Validation of Docking Protocol

The docking protocol was validated by redocking the native ligand (P218) using a grid box defined by its position and dimensions. This grid box, centered on the native ligand, was subsequently used for ligands docking. The poses of the cocrystallized and the redocked native ligands were compared by calculating the root mean square deviation (RMSD). The protocol is considered valid if the RMSD is less than 2.0 [20, 21].

### 2.4. Molecular Docking

Molecular docking was performed using Glide in Schrödinger suite [22]. The procedure began by selecting the prepared *Mu*DHFR as the macromolecule and all ligands to be docked on the Maestro entry list. A receptor grid box was generated, centered around the position of the cocrystallized ligand (P218) with coordinates of *X* = −2.41, *Y* = −2.83, *Z* = 15.92, and a ligand diameter midpoint box of 10 Å in all three coordinates. Flexible ligand sampling with extra precision (XP) was selected to run the molecular docking simulation. The output settings of the docking results were kept at their default values to report the best pose with the highest docking score for each ligand.

### 2.5. Prime MM‐GBSA Calculations

The Top 30 phytochemicals with the highest docking scores were rescored using the Prime MM‐GBSA module [23] to accurately calculate binding free energy (*Δ* *G*) in kilocalories per mole. The calculations were based on the best‐docked pose obtained from Glide XP docking. The system was minimized using the OPLS4 force field within the variable surface generalized born (VSGB 2.0) solvation model [24], and the free energy difference between the bound and unbound states was calculated using the MM‐GBSA method. This approach considers the enthalpic contributions to binding, while implicitly accounting for entropic effects through the sampling of conformational space during the minimization process [24, 25]. Molecules with the highest binding affinities to *Mu*DHFR were selected and prioritized for further ADMET predictions, drug‐likeness analysis, and molecular dynamic simulations.

### 2.6. Pharmacokinetics, Toxicity, and Drug‐Likeness Properties

The lack of pharmacokinetics characterization leads to a high attrition rate in potential drugs as they progress to clinical trials, resulting in delayed drug development and the wastage of limited resources, including funds. In silico systems that help predict pharmacokinetic parameters and drug‐likeness characteristics of molecules are therefore invaluable in minimizing this challenge in drug discovery and development. In this study, we employed the SwissADME web tool (http://www.swissadme.ch/) to evaluate physicochemical properties and drug‐likeness [26], and the Deep‐PK server (https://biosig.lab.uq.edu.au/deeppk/), a deep learning‐based platform [27], to predict a comprehensive suite of pharmacokinetic and toxicity parameters for the Top 6 phytochemicals, the cocrystallized ligand (P218), and the standard drugs methotrexate and trimethoprim.

### 2.7. MD Simulations

To rigorously evaluate the dynamic behavior, interaction stability, and binding affinity of the most promising phytochemicals, extended all‐atom MD simulations were performed. A total of 10 molecular systems were prepared for MD simulations, including the Apo (unbound) *Mu*DHFR structure and its complexes with P218, kaempferol‐3‐O‐rutinoside, neochlorogenic acid, 3‐glucosylmaclurin, methotrexate, procyanidin, trimethoprim, 6‐O‐galloylglucose, and ellagic acid. Prior to simulations, system preparation was conducted using Maestro, where adjustments were made to protonation states, essential hydrogen atoms were corrected, and termini residues were capped to stabilize the protein structure [28–31]. The unbound *Mu*DHFR system was processed using validated simulation protocols employed in previous studies [28, 32, 33].

### 2.8. Simulation Protocol

All MD simulations were executed using the AMBER18 software suite with the PMEMD (Particle Mesh Ewald MD) engine [34, 35]. The protein structures were assigned appropriate protonation states and modeled using the AMBER FF14SB force field [36, 37]. Structural preparation included histidine protonation and renaming using a customized pdb4amber script. Topology and parameter files for *Mu*DHFR were generated using the LEAP module, which also facilitated system neutralization. Energy minimization began with a harmonic restraint of 500 kcal/mol, followed by 2500 steps of restrained and 5000 steps of unrestrained minimization. The system temperature was gradually increased from 0 to 310 K. Pressure equilibration at 1 atm was maintained using a Berendsen barostat, and further equilibration occurred over 1000 ps at 310 K [38]. Each system was then subjected to a 300‐ns MD simulation to analyze the structural effects of the selected ligands on *Mu*DHFR [39].

### 2.9. Post‐MD Simulation Analysis

The simulation trajectories for both the Apo and ligand‐bound *Mu*DHFR systems were analyzed using the CPPTRAJ module in AMBER18, with data recorded at 1 ps intervals [40]. Post‐MD analysis included calculations of structural stability (RMSD), residue‐level flexibility (root mean square fluctuation (RMSF)), molecular compactness (radius of gyration (RoG)), and solvent‐accessible surface area (SASA). To further understand conformational changes, principal component analysis (PCA) was applied to evaluate large‐scale atomic displacements. Additionally, the binding free energies between *Mu*DHFR and other ligands were estimated using the molecular mechanics/generalized born surface area (MM/GBSA) approach. Visualization and analysis of simulation outputs were performed using Microcal Origin [41] and Discovery Studio [42, 43].

### 2.10. Calculations of the Free Energy of Protein–Ligand Interactions Using MM/GBSA

The MM/GBSA method was employed to determine the binding free energy, including van der Waals, electrostatic, and solvation contributions, for each ligand–*Mu*DHFR complex [44–47]. The binding free energy was calculated using the following equations:

(i) ΔGbind=Gcomplex−Gprotein−Ginhibitor,

(ii) ΔGbind=Egas+Gsol−TS.

The *Δ* *G*_bind_ is the sum of the gas and solvent energy minus entropy (TS).

(iii) Egas=Eint+Evdw+Eele.

In this context, *E*_gas_ represents the total internal energy of the AMBER force fields, which include *E*_int_ for bond, torsion, and angle energies; *E*_vdw_ for van der Waals energies related to covalent bonds; and *E*_elec_ for electrostatic energies related to non‐bonded pairs.

The equation used for calculating the solvent energy is as follows:

(iv) Gsol=GGB+GSA,

(v) GSA=γSASA.

*G*_GB_ stands for the polar solvation effect and *G*_SA_ for the nonpolar solvation effect, both calculated using the SASA. This is attainable by the 1.4 Å water probe combined with ‘*b*’ surface tension constant values of 0 kcal/mol and ‘*c*’ values of 0.0072 kcal/mol [48]. Finally, to investigate the stability of *Mu*DHFR, a per‐residue energy decomposition (PRED) analysis was carried out to determine the possible energy contributions of certain residues inside the catalytic region.

### 2.11. PCA

PCA was utilized to explore the dynamic behaviour of *Mu*DHFR in its Apo state and when complexed with each ligand [49]. This method reduces the dimensionality of trajectory data to capture major correlated or anticorrelated motions of the protein structure [50]. The atomic coordinates and eigenvectors were utilized to produce a positional covariance matrix *C*, subsequently employed to analyze collective motions. The eigenvalues indicate the extent of the motion, whereas the eigenvectors indicate the direction of the motion [51]. The positional covariance matrix *C* was calculated using the equation provided:

(1) Ci=qi−qiqj−qji,j=123,,⋯,N.

The variables *q*_*i*_ and *q*_*j*_ represent the Cartesian coordinates of the *i* ^th^ and *j* ^th^ C*α* atoms, respectively, and *N* denotes the total number of C*α* atoms. The average is calculated after superimposing the MD trajectories with a reference structure using a least‐squares fit approach to extract the essential motion, so excluding all translational and rotational movements [52]. The eigenvalues and eigenvectors are computed by performing an orthogonal coordinate transformation on the symmetric matrix *C*, resulting in a diagonal matrix *Λ* of eigenvalues, as demonstrated below:

(2) Λ=TTCijT.

The eigenvalues here indicate the system’s total mean‐square variation along each eigenvector, and the eigenvectors point in the directions of motion to (*q*_*i*_).

## 3. Results and Discussions

### 3.1. Docking Validation

**Figure 1:**
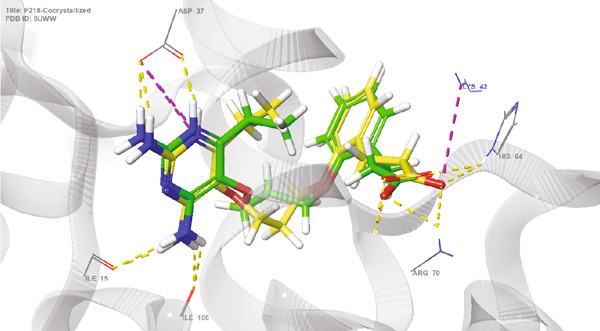
Validation of docking protocol through redocking of native ligand (P218) on *Mu*DHFR target. Superimposed crystallized (yellow) ligand (P218) and redocked (green) ligand (RMSD = 0.89).

The use of redocking in validating docking protocols helps in assessing accuracy and benchmarking performance. When there is a high level of repetition (RMSD < 2) between the experimentally determined and redocked poses, it gives confidence about the predictability of the docking results [20, 21, 53]. Figures 1 and 2 illustrate the superimposed 3D and 2D structures of the cocrystallized ligand (P218) and the redocked ligand, respectively. The calculated RMSD of 0.89 is indicative of the validity of the docking protocol and therefore a high degree of confidence in molecular docking results.

Figure 2Comparison of binding poses and ligand–protein interactions at the *Mu*DHFR active site: (a) redocked ligand and (b) native cocrystallized ligand. The similarity in interactions with key active‐site residues confirms the success of the docking protocol.

**Figure:**
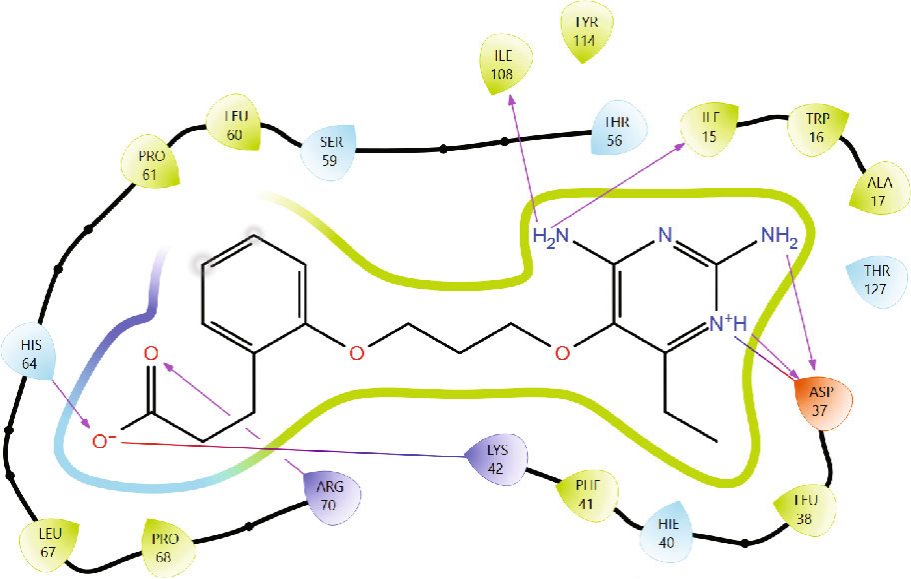
(a)

**Figure:**
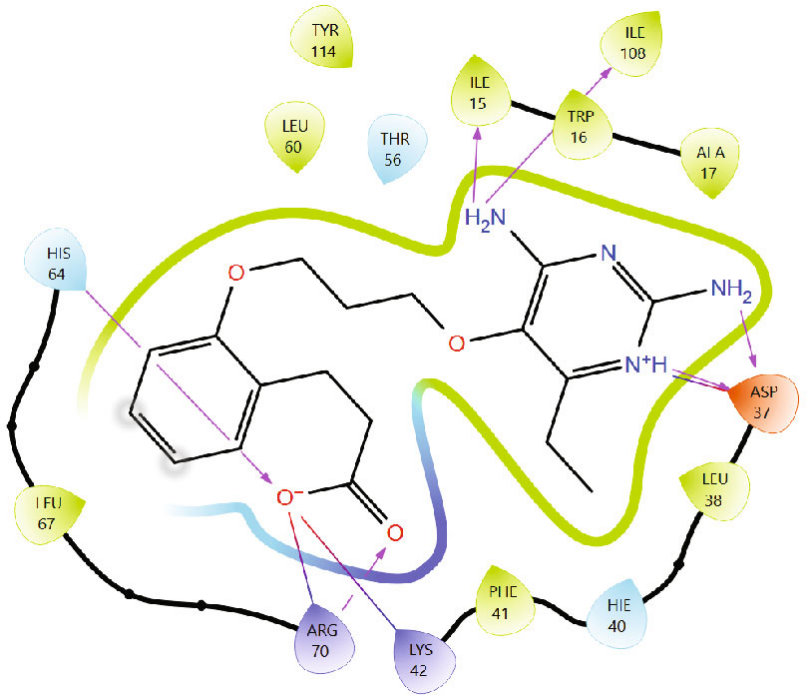
(b)

### 3.2. Molecular Docking

Molecular docking using Glide XP successfully identified a range of phytochemicals with favorable binding poses within the active site of *Mu*DHFR. This initial screening served as a crucial first step to narrow down the vast chemical space of the 172 phytochemicals to a manageable number of high‐potential candidates for more computationally intensive evaluations.

Glide successfully docked 155 out of the 172 phytochemicals (90.0%) against the *Mu*DHFR active site. The remaining 17 ligands were skipped by Glide due to various issues, such as failure to identify suitable binding poses, unsuccessful grid energy minimization, or breakdown during rough pose refinement. Of the successfully docked ligands, 142 (91.6%) exhibited negative Glide XP docking scores, ranging from −13.60 to −0.09 kcal/mol, indicating favorable interactions with the target protein. In contrast, 13 ligands (8.4%) showed positive docking scores, suggesting unfavorable binding. The Top 30 phytochemicals were selected for further analysis based on a combination of criteria: favorable Glide XP docking scores, more negative Prime MM‐GBSA *Δ* *G*_bind_ values (reflecting stronger binding affinity), and representation across all screened plant species to ensure chemical diversity. Among the selected compounds, the majority (76.6%) originated from *M. indica*, followed by *N. tabacum* (10%), with *C. papaya* and *Solanum torvum* each contributing approximately 6.7%. While this provided an initial refinement of binding affinity estimates, such single‐pose calculations cannot account for protein flexibility or the dynamic nature of ligand binding. Recognizing this limitation, we proceeded with extensive MD simulations to provide a more accurate and reliable assessment of binding and stability.

Table 1 presents the docking scores and binding affinities (Prime MM‐GBSA *Δ* *G*_bind_) of the Top 30 phytochemicals, along with the cocrystallized ligand P218 and two standard DHFR inhibitors, methotrexate and trimethoprim. The complete docking and MM‐GBSA results for all compounds are provided in Table S2.

**Table 1:** Docking scores, MM‐GBSA binding affinities to *Mu*DHFR, and origins of the Top 30 phytochemicals compared to three reference compounds (P218, trimethoprim, and methotrexate). Compounds are arranged based on their MM‐GBSA scores.

**No.**	**Compound**	**Origin**	**Docking score**	**MM-GBSA,** **Δ** **G** **(kcal/mol)**
R_1_	**P218**	**Cocrystallized ligand**	**−11.88**	**−70.5**
R_2_	**Trimethoprim**	**Standard drug**	**−9.29**	**−64.39**
1	6‐O‐Galloylglucose	*Mangifera indica*	−9.99	−64.06
R_3_	**Methotrexate**	**Standard drug**	**−10.04**	**−58.63**
2	3‐Glucosylmaclurin	*Mangifera indica*	−10.62	−56.03
3	Procyanidin	*Mangifera indica*	−11.14	−54.17
4	Ellagic acid	*Mangifera indica*	−8.16	−53.31
5	Kaempferol‐3‐O‐rutinoside	*Nicotiana tabacum*	−11.23	−53.2
6	Neochlorogenic acid	*Nicotiana tabacum*	−9.06	−52.57
7	Luteolin 7‐O‐glucoside	*Mangifera indica*	−10.08	−52.4
8	Rosmarinic acid	*Mangifera indica*	−8.25	−50.53
9	Epigallocatechin Gallate	*Mangifera indica*	−8.61	−50.46
10	Isovitexin	*Mangifera indica*	−8.85	−50.3
11	Rutin	*Nicotiana tabacum*	−11.8	−48.05
12	Myricetin	*Mangifera indica*	−8.88	−47.09
13	Mangiferin	*Mangifera indica*	−9.29	−44.92
14	Tetra‐o‐galloylglucose	*Mangifera indica*	−13.6	−44.68
15	Vitexin	*Mangifera indica*	−9.8	−42.98
16	Taxifolin	*Mangifera indica*	−8.07	−42.77
17	Torvoside E	*Solanum torvum*	−9.19	−42.16
18	Iriflophenone 3‐C‐glucoside	*Mangifera indica*	−9.65	−42.03
19	Hyperoside	*Mangifera indica*	−9.31	−41.72
20	Torvanol A	*Solanum torvum*	−8.32	−40.35
21	Isomangiferin	*Mangifera indica*	−10.46	−39.87
22	Apigetrin	*Mangifera indica*	−9.65	−39.24
23	Quercetin‐3‐glucoside	*Mangifera indica*	−9.17	−39.14
24	Beta‐glucogallin	*Mangifera indica*	−8.32	−38.14
25	Riboflavin	*Carica papaya*	−9.27	−37.65
26	Indolylglucosinolate	*Carica papaya*	−10.88	−35.6
27	Quercetin‐3‐O‐deoxyhexosyl (1‐2) pentoside	*Mangifera indica*	−10.39	−32.14
28	Apigenin 7‐O‐diglucuronide	*Mangifera indica*	−10.34	−26.63
29	Querciturone	*Mangifera indica*	−8.89	−24.89
30	Isoquercitin	*Mangifera indica*	−10.41	−24.51

*Note:* Bold entries represent reference drugs for easy comparison.

**Figure 3:**
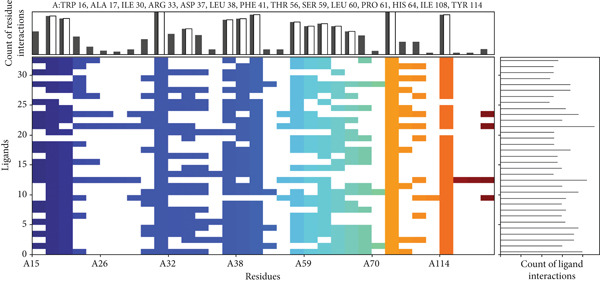
Ligand–protein interaction fingerprints for the Top 30 docked compounds at the *Mu*DHFR binding site. The heat map summarizes key interactions between each ligand and the binding site residues, as generated by Maestro (Schrödinger). Each row represents a compound, while each column corresponds to an amino acid residue within the binding site. The color‐coded cells indicate the presence or absence of interactions. Colored cells denote the presence of an interaction, while blank cells indicate absence. The interaction pattern highlights residues frequently involved in ligand binding and allows for visual comparison of binding profiles across the compound set.

**Figure 5:**
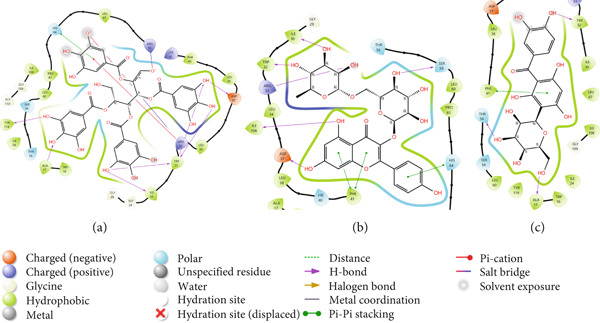
Protein–ligand interactions for (a) tetra‐O‐galloylglucose, (b) kaempferol‐O‐rutinoside, and (c) 3‐glucosylmaclurin with *Mu*DHFR.

Comparison of the protein–ligand interactions between *Mu*DHFR and its cocrystallized ligand (P218), the standard drugs (methotrexate and trimethoprim), or with the phytochemicals revealed commonalities in the nature of interactions and the identity of the interacting residues in the binding site. As illustrated by interaction fingerprints (Figure 3), the most common residues were ILE 15, ALA 17, ILE 30, ARG 33, ASP 37, LEU 38, PHE 41, THR 56, SER 59, LEU 60, HIS 64, ILE 108, and TYR 114. In general, the molecules examined exhibited diverse interactions with the *Mu*DHFR target through important residues, such as ILE 15, ASP 37, PHE 41, THR 56, SER 59, HIS 64, and ILE 108, as shown in Figures 4, 5, 6, and 7. Stable interactions through these critical residues are likely to cause inhibition of the *Mu*DHFR enzyme.

Figure 4Protein–ligand interactions of (a) methotrexate, (b) trimethoprim, and (c) P218.

**Figure:**
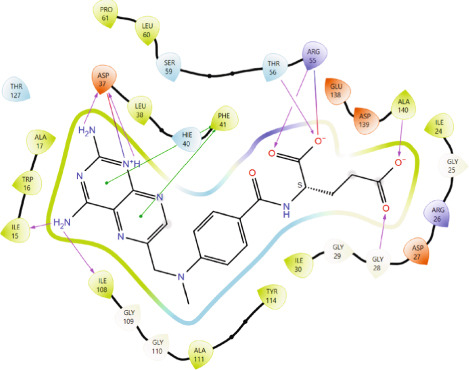
(a)

**Figure:**
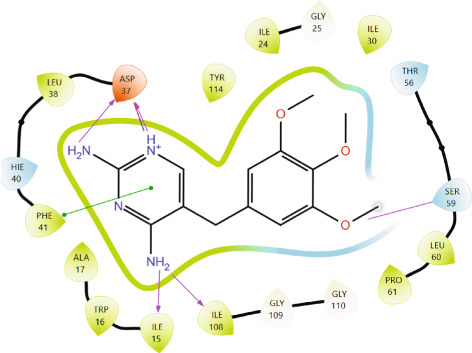
(b)

**Figure:**
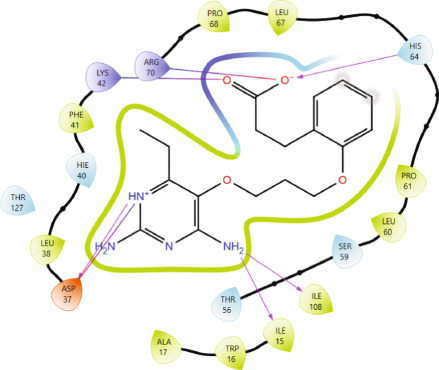
(c)

Figure 6Protein–ligand interactions of (a) 6‐O‐galloylglucose and (b) neochlorogenic acid with *Mu*DHFR.

**Figure:**
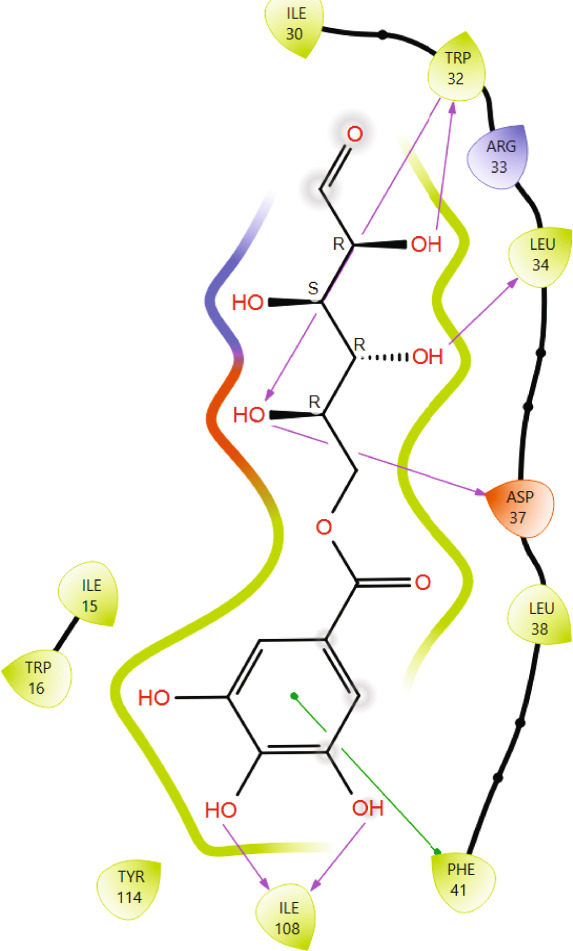
(a)

**Figure:**
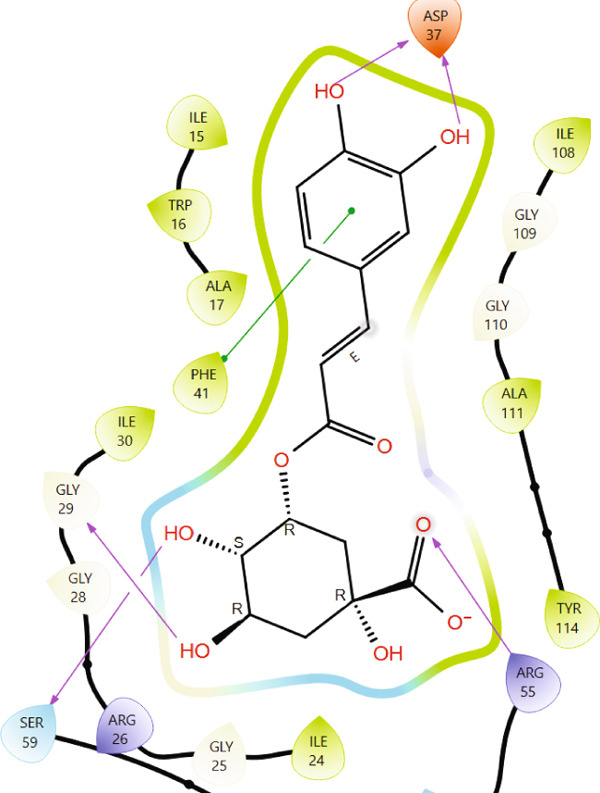
(b)

Figure 7Protein–ligand interactions of (a) procyanidin and (b) ellagic acid with *Mu*DHFR.

**Figure:**
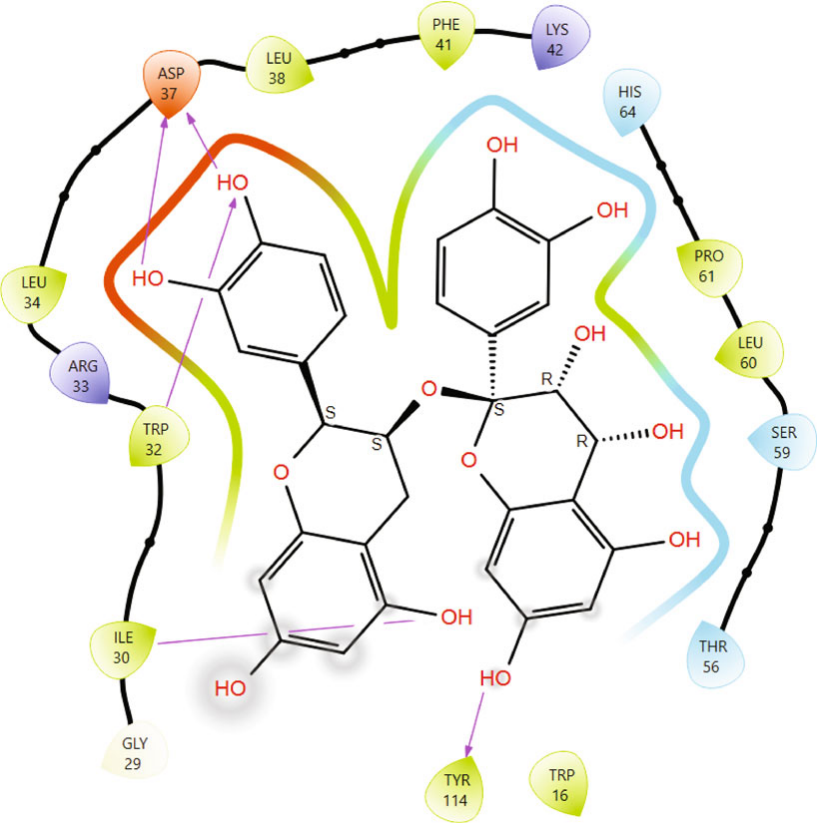
(a)

**Figure:**
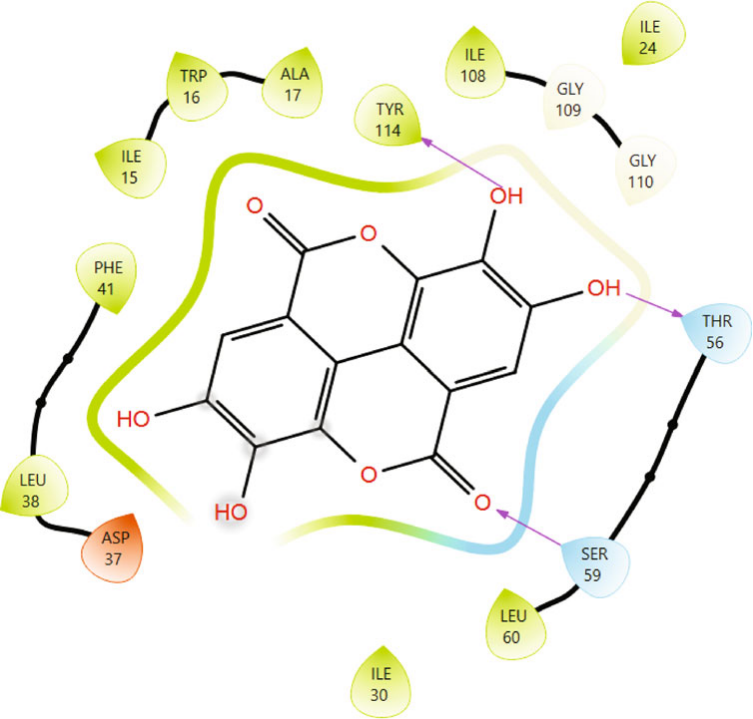
(b)

### 3.3. Prime MM‐GBSA Calculations

The MM/GBSA method combines molecular mechanics force fields (MM) with implicit solvation models (generalized born and surface area (GBSA)) to estimate the binding free energy (*Δ* *G*) [24]. As summarized in Table 2, the cocrystallized ligand (P218) showed the highest *Δ* *G* (−70.50 kcal/mol) followed by trimethoprim (−64.39 kcal/mol). Among the Top 30 molecules, 6‐O‐galloylglucose had the highest binding free energy (−64.06 kcal/mol), not far behind trimethoprim but higher than that of methotrexate (−58.63 kcal/mol). Out of the Top 6 molecules (Table 2) with the highest binding free energy to *Mu*DHFR, four (6‐O‐galloylglucose, 3‐glucosylmaclurin, procyanidin, and ellagic acid) were isolated from *M. indica* and two (kaempferol‐3‐O‐rutinoside and neochlorogenic acid) from *N. tabacum*.

**Table 2:** Top 6 phytochemicals with the highest binding affinities.

**No.**	**Compound**	**Origin**	**Docking score**	**MM-GBSA,** **Δ** **G** **(kcal/mol)**
R_1_	**P218**	**Cocrystallized ligand**	−11.88	−70.5
R_2_	**Trimethoprim**	**Standard drug**	−9.29	−64.39
1	6‐O‐Galloylglucose	*Mangifera indica*	−9.99	−64.06
R_3_	**Methotrexate**	**Standard drug**	−10.04	−58.63
2	3‐Glucosylmaclurin	*Mangifera indica*	−10.62	−56.03
3	Procyanidin	*Mangifera indica*	−11.14	−54.17
4	Ellagic Acid	*Mangifera indica*	−8.16	−53.31
5	Kaempferol‐3‐O‐rutinoside	*Nicotiana tabacum*	−11.23	−53.2
6	Neochlorogenic acid	*Nicotiana tabacum*	−9.06	−52.57

*Note:* Bold entries represent reference drugs for easy comparison.

It is important to point out that docking scores and MM‐GBSA are two different methods for evaluating ligand–protein binding affinity. Docking scores employ empirical scoring functions to estimate binding affinity based on generated poses. Although docking scores are fast and suitable for the initial screening of large ligand libraries, they are less accurate in predicting absolute binding affinity [54, 55]. Molecular docking relies on scoring functions to assess the quality of the predicted binding poses generated during conformational search. These functions utilize approximations to streamline the calculations, enabling high‐throughput screening of potential drug candidates. Due to the use of approximate scoring functions, molecular docking often falls short of accurately predicting binding energies when compared to experimental measurements. Although numerous docking programs effectively identify potential ligand binding conformations, a universal scoring function that accurately predicts binding energies for all molecules and protein families remains elusive. Consequently, rescoring steps after molecular docking are often essential to refine the initial predictions [56].

On the other hand, MM‐GBSA utilizes molecular mechanics and free energy calculations to consider enthalpic contributions and thus provide more accurate binding affinity predictions [54]. MM‐GBSA is computationally more expensive but offers greater accuracy and insights into binding mechanisms, making it ideal for ranking and refining promising ligands [54, 57]. Instead of simulating the MD of a system over time, the Prime MM‐GBSA module in the Schrödinger suite performs a series of calculations on a single optimized pose from Glide’s docking output, using a generalized born solvation model (VSGB 2.0) [24] to estimate the binding free energy. These calculations involve minimization to relax any strained interactions, energy calculation of the complex, and its individual components using the MM‐GBSA method, and finally, determining the binding affinity by calculating the difference in energies (*E*) of the protein–ligand’s complex and its individual components [58].

ΔGbind=E_complexminimized–E_ligandminimized–E_receptorminimized.

The Prime MM‐GBSA approach offers significant time efficiency compared to standard MM‐GBSA, which relies on lengthy MD simulations [56]. However, the Prime MM‐GBSA method is limited by the single, optimized docked pose, potentially missing the full range of conformations the ligand can adopt in the binding site and potentially neglecting entropic effects [59]. Overall, Prime MM‐GBSA can be a useful tool for quick binding affinity estimations [60], but a full MD simulation provides a more accurate and complete understanding of the protein–ligand binding process. Therefore, to overcome this limitation and to get a more accurate estimation of binding free energy, we performed an MD simulation for the Top 6 hit compounds and then recalculated MM‐GBSA for the different conformations that the ligands would adopt over the simulation time.

### 3.4. *In Silico* Pharmacokinetic and Toxicity Profiling

To mitigate the high attrition rates common in later stages of drug development, the early *in silico* assessment of absorption, distribution, metabolism, excretion, and toxicity (ADMET) properties provides crucial guidance for prioritizing lead candidates. It is important to recognize that these computational predictions serve as valuable screening tools for hypothesis generation and must be interpreted with caution, as they do not replace the need for future experimental validation.

### 3.5. Physicochemical Properties and Drug‐Likeness

The drug‐likeness of the compounds was initially assessed using the established rules of Lipinski and Veber, which are primarily calibrated for orally bioavailable drugs. These rules suggest that drug‐like molecules should generally possess a molecular weight (MW) ≤ 500 g/mol, a calculated logarithm of the partition coefficient (Log*p*) ≤ 5, five or fewer hydrogen bond donors (HBDs), not more than 10 hydrogen bond acceptors (HBA), a topological polar surface area (TPSA) ≤ 140 Å^2^, and ≤ 10 rotatable bonds (RBs) [61, 62].

As detailed in Table 3, the standard drug trimethoprim and the native ligand P218 adhere to all Lipinski and Veber criteria. In contrast, methotrexate shows violations, primarily related to its high number of O and N atoms, or polar surface area. The phytochemicals, which are predominantly large polyphenolic glycosides, exhibit multiple violations of these rules, particularly concerning MW, HBD, HBA, and TPSA. For instance, kaempferol‐3‐O‐rutinoside and procyanidin have MW values approaching 600 g/mol and TPSA values well over 200 Å^2^. These violations are expected for this class of natural products and are consistent with their known poor oral bioavailability. However, the “drug‐likeness” paradigm defined by Lipinski’s Rule of Five is not a rigid filter, and numerous approved drugs, particularly natural products and transporter substrates, deviate from these guidelines [63, 64]. More critically, for the therapeutic context of BU, a cutaneous disease, the intended application of these compounds is topical. Therefore, properties governing oral absorption are of secondary importance, and strict adherence to these rules is not a prerequisite for efficacy or safety in this specific application.

**Table 3:** Drug‐likeness and lead‐likeness predictions from SwissADME.

**Compound**	**MW (g/mol)**	**L** **o** **g** **p**	**#HBA**	**#HBD**	**TPSA**	**#RB**	**Number of violations**		
							**Lipinski**	**Veber**	**Lead-likeness**
P218	360.41	1.07	5	3	137.66	10	0	0	2
Kaempferol‐3‐O‐rutinoside	594.52	−3.43	15	9	249.2	6	3	1	1
Procyanidin	594.52	−0.6	13	10	229.99	4	3	1	1
3‐Glucosylmaclurin	424.36	−2.77	11	9	208.37	4	2	1	1
Methotrexate	454.44	−0.73	9	5	210.54	10	1	1	2
6‐O‐Galloylglucose	332.26	−2.39	10	7	184.98	8	1	1	1
Trimethoprim	291.33	0.41	4	3	106.76	5	0	0	0
Neochlorogenic acid	353.3	−1.05	9	5	167.58	5	0	1	1
Ellagic acid	302.19	0.14	8	4	141.34	0	0	1	0

*Note:* Reference values: Lipinski: MW ≤ 500, CLog*p* ≤ 5, HBA ≤ 10, and HBD ≤ 5; Veber: TPSA ≤ 140, RB ≤ 10; and Lead‐likeness: MW = 250–350, RB ≤ 7, Log*p* ≤ 3.5.

### 3.6. Pharmacokinetic Properties: ADME

#### 3.6.1. Absorption and Distribution

The predicted human intestinal absorption for all six phytochemicals was low, with most compounds predicted to be “nonabsorbed” (Table 4). This finding aligns with their physicochemical properties (high MW and polarity) and reinforces the unsuitability of an oral route of administration.

**Table 4:** Predicted absorption and distribution properties.

**Compound**	**Human intestinal absorption**	**Skin permeability (**log **K** **p** **, cm/s)**	**P-gp substrate**	**P-gp inhibitor**	**BBB permeable**
P218	Absorbed	−3.06	No	No	Yes
Kaempferol‐3‐O‐rutinoside	Nonabsorbed	6.18	Yes	No	No
Procyanidin	Nonabsorbed	12.68	Yes	Yes	No
3‐Glucosylmaclurin	Nonabsorbed	2.05	No	No	No
Methotrexate	Absorbed	−2.46	No	No	Yes
6‐O‐Galloylglucose	Nonabsorbed	−0.72	No	No	No
Trimethoprim	Absorbed	−2.55	No	No	Yes
Neochlorogenic acid	Absorbed	−2.75	No	No	No
Ellagic acid	Absorbed	−0.72	No	No	No

Of paramount importance for a topical therapy is skin permeability, which dictates the extent to which a compound can penetrate the stratum corneum to reach its site of action while minimizing leakage into systemic circulation [65]. The predicted skin permeability, expressed as log *K* *p* (cm/s), was low for all phytochemicals. Notably, compounds like kaempferol‐3‐O‐rutinoside (log *K* *p* = 6.18) and procyanidin (log *K* *p* = 12.68) have extremely low predicted permeability values. This is a highly favorable characteristic for a topical agent intended for local action, as it suggests the compounds are likely to remain concentrated in the dermal layers where the *M. ulcerans* infection resides, thereby reducing the potential for systemic side effects. All compounds were also predicted to be non‐penetrable across the blood–brain barrier (BBB), further indicating a low risk of central nervous system effects should any systemic absorption occur.

P‐glycoprotein (P‐gp) is a key efflux transporter that can limit the bioavailability of xenobiotics [66]. The predictions indicate that most of the phytochemicals are not P‐gp inhibitors, with the exception of procyanidin. Kaempferol‐3‐O‐rutinoside and procyanidin were predicted to be P‐gp substrates, suggesting that even if they were to enter cells, an active efflux mechanism may be present.

#### 3.6.2. Metabolism and Excretion

The interaction of the compounds with the cytochrome P450 (CYP) enzyme system is critical for assessing potential drug–drug interactions. CYP450 enzymes are essential in drug and xenobiotic metabolism. They account for about 80% of the biotransformation of all drugs in clinical use [67, 68]. As shown in Table 5, none of the Top 6 phytochemicals were predicted to inhibit CYP2D6 or CYP3A4, the primary isoenzymes involved in metabolizing approximately 52% of all drugs [69]. Procyanidin was flagged as a potential inhibitor of CYP2C9, and ellagic acid was predicted to inhibit CYP1A2, CYP2C9, and CYP2C19. However, given the low predicted systemic exposure via topical application, the clinical significance of these potential interactions is likely minimal.

**Table 5:** Predicted metabolism and excretion properties.

**Compound**	**CYP1A2 inhibitor**	**CYP2C9 inhibitor**	**CYP2C19 inhibitor**	**CYP2D6 inhibitor**	**CYP3A4 inhibitor**	**Total clearance (log mL/min/kg)**	**T**_1/2_ **(h)**
P218	No	No	No	No	No	4.57	< 3
Kaempferol‐3‐O‐rutinoside	No	No	No	No	No	11.92	< 3
Procyanidin	No	Yes	No	No	No	12.56	< 3
3‐Glucosylmaclurin	No	No	No	No	No	12	< 3
Methotrexate	No	No	No	No	No	0.6	< 3
6‐O‐Galloylglucose	No	No	No	No	No	11.84	≥ 3
Trimethoprim	No	No	No	No	No	5.22	< 3
Neochlorogenic acid	No	No	No	No	No	6.54	≥ 3
Ellagic acid	Yes	Yes	Yes	No	No	7.7	< 3

The predicted total clearance and half‐life (*T*_1/2_) provide an estimate of how quickly the compounds would be eliminated if they reached systemic circulation. Most compounds were predicted to have a short half‐life (< 3 h), suggesting rapid elimination and low potential for accumulation. While these parameters are included for completeness, they are of less concern for a topical medication where the primary goal is local, rather than systemic, therapeutic concentrations.

### 3.7. Toxicity Profile

Toxicity profiles of the compounds were evaluated and presented in Table 6.

**Table 6:** Predicted toxicity profile.

**Compound**	**Skin sensitization**	**AMES mutagenicity**	**HERG I inhibitor**	**Hepatotoxicity (DILI)**	**MTD (log mg/kg/day)**	**Rat oral LD**_**50**_ **(predicted)**
P218	Safe	Safe	Safe	Toxic	0.24	Toxic
Kaempferol‐3‐O‐rutinoside	Safe	Safe	Safe	Safe	0.94	Toxic
Procyanidin	Safe	Safe	Safe	Toxic	1.51	Toxic
3‐Glucosylmaclurin	Safe	Toxic	Safe	Safe	0.70	Toxic
Methotrexate	Safe	Safe	Safe	Toxic	‐0.73	Toxic
6‐O‐Galloylglucose	Safe	Safe	Safe	Safe	1.16	Safe
Trimethoprim	Safe	Safe	Toxic	Toxic	0.38	Toxic
Neochlorogenic acid	Safe	Safe	Safe	Safe	0.69	Safe
Ellagic acid	Safe	Safe	Safe	Toxic	1.14	Toxic

Abbreviations: DILI: drug‐induced liver injury; MTD, maximum tolerated dose.

#### 3.7.1. Dermal and Local Toxicity

For any topically applied agent, the potential to cause skin sensitization is a primary safety concern. Encouragingly, all six phytochemicals, as well as the standard drugs, were predicted to be nonsensitizers. This is a critical and highly favorable finding that strongly supports their potential for development as topical treatments for BU.

#### 3.7.2. Systemic Toxicity

Acute systemic toxicity is often assessed by the oral lethal dose 50 (LD_50_) [70]. *In silico* LD_50_ prediction can be unreliable. The Deep‐PK model predicts toxicity as a classification (e.g., “Toxic” or “Safe”) rather than a precise quantitative value. To transparently assess the model’s performance and contextualize our predictions, we compared the model’s output for our standard drugs with their published experimental oral LD_50_ values in rats. The experimental oral LD_50_ for methotrexate is approximately 135 mg/kg [71], while for trimethoprim, it is > 2000 mg/kg [72], indicating methotrexate is significantly more toxic via the oral route. The Deep‐PK model correctly classifies both as “Toxic” but fails to capture this large quantitative difference, highlighting the limitations of current computational models for this specific endpoint and chemical space. Given these limitations and the intended topical route of administration, the predicted systemic toxicities must be interpreted with caution and in the context of low systemic exposure.

#### 3.7.3. hERG Inhibition

Blockade of the hERG (human Ether‐a‐go‐go‐Related Gene) potassium channel is a major cause of drug‐induced cardiotoxicity [73]. The Deep‐PK model predicts that all six phytochemicals are not hERG blockers. This favorable prediction is mechanistically plausible, as these polyphenols lack the key structural features—typically a basic nitrogen and high lipophilicity—common to most hERG inhibitors [74].

#### 3.7.4. Mutagenicity (AMES Test)

The Deep‐PK model predicted that 3‐glucosylmaclurin may be mutagenic. This is a noted liability for some flavonoid‐like structures, which can be metabolically activated to reactive species [75]. While this warrants experimental investigation, the clinical risk is likely low due to the compound’s poor predicted skin permeability, which would limit the systemic exposure necessary for hepatic metabolic activation.

#### 3.7.5. Hepatotoxicity

The model predicted a potential for drug‐induced liver injury (DILI) for most of the tested compounds, including the standards. As with other systemic toxicities, this risk is contingent upon significant systemic absorption and liver exposure, which is not anticipated with topical application of these poorly permeable molecules.

In summary, the comprehensive ADMET and toxicity analysis, reframed for the context of a topical therapy, presents a promising profile for the investigated phytochemicals. The most critical safety parameters for this application—skin sensitization and skin permeability—are highly favorable. While some potential systemic toxicities are flagged by the *in silico* models, the risk is substantially mitigated by the low predicted systemic exposure. These findings strongly support the continued investigation of these compounds as potential leads for a locally acting treatment for BU.

### 3.8. MD Simulations

#### 3.8.1. Binding Free Energy Analysis Identified New Lead Candidates

The MM/GBSA method was used to calculate the average binding free energy (*Δ* *G*_bind_) from the 300‐ns simulation trajectories, providing a dynamically averaged measure of binding affinity. The results, summarized in Table 7, revealed a significant reranking of the candidate inhibitors compared to the initial docking scores and Prime MM‐GBSA estimations. Among the tested compounds, the cocrystallized ligand (P218) displayed the most favorable binding affinity (as predicted previously in the docking screening), with a binding free energy *Δ* *G*_bind_ (−59.73 kcal/mol). In contrast, ellagic acid showed the least favorable binding, with a *Δ* *G*_bind_ (−28.41 kcal/mol). The reference drugs, methotrexate and trimethoprim, exhibited *Δ* *G*_bind_ values of (−44.18 kcal/mol) and (−41.01 kcal/mol), respectively. Interestingly, kaempferol‐3‐O‐rutinoside *Δ* *G*_bind_ (−51.62 kcal/mol), neochlorogenic acid *Δ* *G*_bind_ (−49.44 kcal/mol), and 3‐glucosylmaclurin *Δ* *G*_bind_ (−45.19 kcal/mol) demonstrated stronger binding than both standard drugs. Procyanidin also showed a binding energy *Δ* *G*_bind_ (−43.69 kcal/mol) exceeding that of trimethoprim but slightly lower than methotrexate.

**Table 7:** Post‐MD simulations MM/GBSA‐based average binding free energy profiles of various ligands when bound to *Mu*DHFR.

**Systems**	**Energy components (kcal/mol)**				
**Ligand**	**Δ** **E**_**v** **d** **w**_	**Δ** **E**_**e** **l** **e**_	**Δ** **G**_**g** **a** **s**_	**Δ** **G**_**s** **o** **l**_	**Δ** **G**_**b** **i** **n** **d**_
P218	−38.60 ± 0.10	−218.75 ± 0.51	−257.35 ± 0.51	197.63 ± 0.40	−59.73 ± 0.16
Kaempferol‐3‐O‐rutinoside	−56.21 ± 0.09	−39.28 ± 0.18	−95.49 ± 0.21	43.87 ± 0.12	−51.62 ± 0.13
Neochlorogenic acid	−38.22 ± 0.13	−102.49 ± 0.54	−140.70 ± 0.59	91.27 ± 0.43	−49.44 ± 0.24
3‐Glucosylmaclurin	−42.46 ± 0.09	−56.73 ± 0.22	−99.19 ± 0.22	54.00 ± 0.17	−45.19 ± 0.09
Methotrexate	−55.31 ± 0.11	−45.70 ± 0.26	−101.00 ± 0.30	56.82 ± 0.23	−44.18 ± 0.11
Procyanidin	−56.56 ± 0.14	−45.18 ± 0.21	−101.74 ± 0.25	58.04 ± 0.15	−43.69 ± 0.16
Trimethoprim	−36.79 ± 0.07	−94.99 ± 0.23	−131.78 ± 0.23	90.78 ± 0.19	−41.01 ± 0.08
6‐O‐Galloylglucose	−32.44 ± 0.12	−50.63 ± 0.31	−83.06 ± 0.36	53.39 ± 0.23	−29.68 ± 0.16
Ellagic acid	−34.25 ± 0.08	−23.99 ± 0.32	−58.24 ± 0.31	29.83 ± 0.25	−28.41 ± 0.11

*Note:* All energies are in kilocalories per mole.

Abbreviations: *Δ* *E*_ele_, electrostatic energy; *Δ* *E*_vdw_, van der Waals energy; *Δ* *G*_bind_, total binding free energy; *Δ* *G*_gas_ = gas phase free energy; *Δ* *G*_sol_ = solvation free energy.

#### 3.8.2. Per‐Residue Binding Contribution Analysis

Decomposing the total binding energy into individual residue contributions offers detailed insights into the molecular interactions governing ligand binding. To this end, a PRED method was employed to analyze how specific amino acids in the *Mu*DHFR active site contribute to the binding of each compound listed in Table 7. Contributions were assessed by calculating the cumulative effects of total, electrostatic, and van der Waals energies. It is important to point out that due to differences in residue numbering between Schrödinger (used for docking) and AMBER (used for MD simulations), the residue indices in the MD trajectories are offset by 10 residues relative to the initial docking structure. For instance, ARG16 in the AMBER MD output corresponds to ARG26 in the original Schrödinger‐prepared structure. This discrepancy arises because AMBER renumbers residues sequentially starting from the first resolved residue in the PDB structure, while Schrödinger retains the original biological residue numbering from the full‐length protein sequence (PDB ID: 6UWW).

In the P218–*Mu*DHFR complex, ARG23 and ARG60 emerged as the key residues, contributing −163.00 kcal/mol and −172.50 kcal/mol, respectively (Figure 8a). A comparable pattern was observed with kaempferol‐3‐O‐rutinoside, where ARG23 contributed −164.27 kcal/mol (Figure 8b). For neochlorogenic acid, ARG16, ARG23, and ARG45 stood out, with energy contributions of −178.91 kcal/mol, −163.27 kcal/mol, and −174.58 kcal/mol, respectively (Figure 8c).

Figure 8Graphical representation of per‐residue energy contribution of binding site residues of (a) P218, (b) kaempferol‐3‐O‐rutinoside, and (c) neochlorogenic acid towards *Mu*DHFR, respectively. Corresponding intermolecular interactions are displayed: (a) P218, (b) kaempferol‐3‐O‐rutinoside, and (c) neochlorogenic acid.

**Figure:**
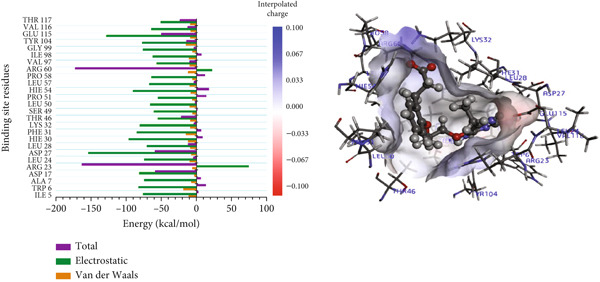
(a)

**Figure:**
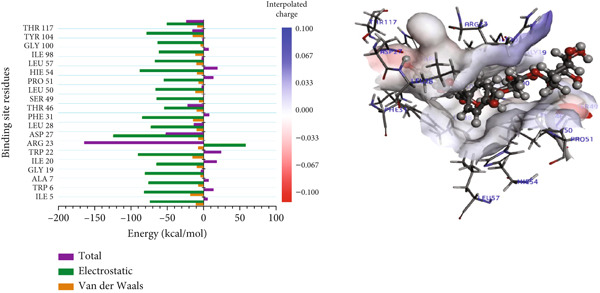
(b)

**Figure:**
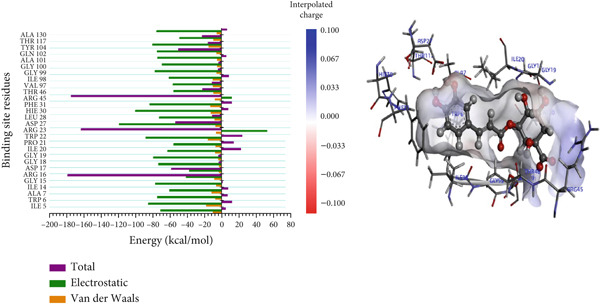
(c)

In the complexes of 3‐glucosylmaclurin and methotrexate with *Mu*DHFR, ARG16, ARG23, and ARG45 again played central roles. Specifically, in the 3‐glucosylmaclurin complex, these residues contributed −179.30, −163.52, and −172.84 kcal/mol, respectively (Figure 9a), while in the methotrexate complex, their contributions were −179.41, −162.05, and −173.05 kcal/mol (Figure 9b). Similarly, procyanidin interacted most strongly with ARG23 (−165.32 kcal/mol) and ARG60 (−162.35 kcal/mol), as shown in Figure 9c.

Figure 9Graphical representation of per‐residue energy contribution of binding site residues of (a) 3‐glucosylmaclurin, (b) methotrexate, and (c) procyanidin towards *Mu*DHFR, respectively. Corresponding intermolecular interactions are displayed: (a) 3‐glucosylmaclurin, (b) methotrexate, and (c) procyanidin.

**Figure:**
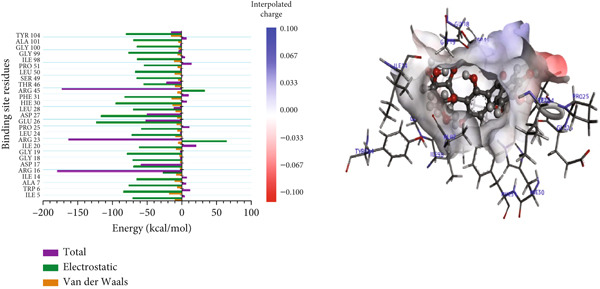
(a)

**Figure:**
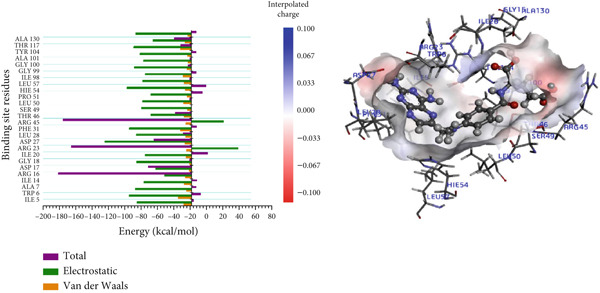
(b)

**Figure:**
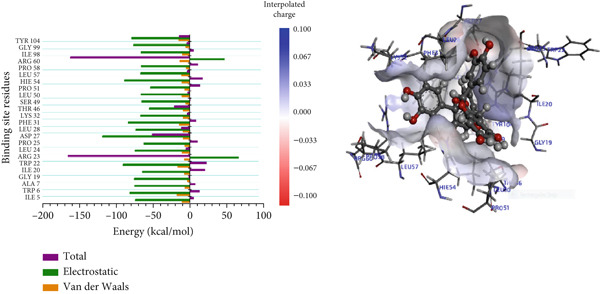
(c)

For the trimethoprim–*Mu*DHFR interaction, ARG60 exhibited the highest binding contribution (−162.16 kcal/mol), while in the 6‐O‐galloylglucose–*Mu*DHFR complex, both ARG23 and ARG60 showed significant energy contributions of −165.43 kcal/mol and −162.91 kcal/mol, respectively (Figure 10a,b). Overall, these findings underscore the critical role of arginine residues—particularly ARG16, ARG23, ARG45, and ARG60—in stabilizing ligand interactions within the *Mu*DHFR binding site.

Figure 10Graphical representation of per‐residue energy contribution of binding site residues of (a) trimethoprim, (b) 6‐O‐galloylglucose, and (c) ellagic acid towards *Mu*DHFR, respectively. Corresponding intermolecular interactions are displayed: (a) trimethoprim, (b) 6‐O‐galloylglucose, and (c) ellagic acid.

**Figure:**
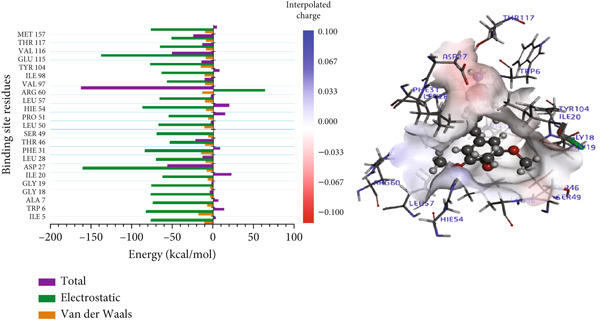
(a)

**Figure:**
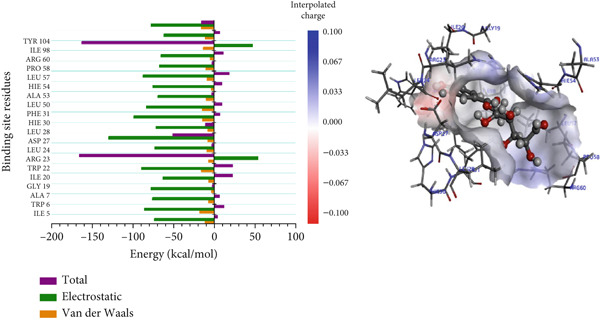
(b)

**Figure:**
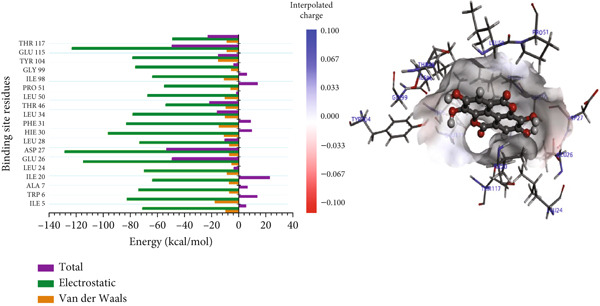
(c)

#### 3.8.3. Analysis of the Structural Landscapes Associated With the Binding of Ligands to *Mu*DHFR

Proteins are inherently responsive to external stimuli, with the binding of small molecules often triggering structural adaptations [76]. These conformational shifts are directly tied to the protein’s function, making them critical for understanding how inhibitors modulate biological activity [77]. Because many therapeutic compounds exert their effects by interacting with and altering protein structures implicated in disease, analyzing these structural modifications is essential. In this context, we investigated how the binding of several ligands affects the structural behavior of *Mu*DHFR. To initiate this analysis, we assessed the structural stability of *Mu*DHFR in both its unbound (Apo) form and when complexed with the aforementioned ligands using RMSD to ensure simulation equilibration. Complementary structural insights were obtained RMSF and RoG metrics, which are informative of residue flexibility and molecular compactness, respectively. RMSF quantifies the degree of fluctuation among atoms, reflecting local flexibility [78], while RoG provides an overview of the spatial distribution and folding of the protein structure [79]. Additionally, SASA was used to predict protein folding tendencies and surface exposure [80], offering further clues about structural stability through the interaction of hydrophobic and hydrophilic residues.

Figure 11a and Table 8 reveal average RMSD values for *Mu*DHFR in both unbound and ligand‐bound states. Compounds such as P218, kaempferol‐3‐O‐rutinoside, methotrexate, procyanidin, and trimethoprim were associated with reduced backbone deviations compared to the Apo form, indicating increased stabilization. In contrast, neochlorogenic acid, 3‐glucosylmaclurin, 6‐O‐galloylglucose, and ellagic acid induced greater deviations, suggesting structural destabilization or flexibility upon binding. RMSF data (Figure 11b and Table 8) further emphasized these trends. Notably, trimethoprim lowered the flexibility of *Mu*DHFR residues relative to the Apo state, as shown by reduced RMSF values. The other ligands—P218, kaempferol‐3‐O‐rutinoside, neochlorogenic acid, 3‐glucosylmaclurin, methotrexate, procyanidin, 6‐O‐galloylglucose, and ellagic acid—increased residue fluctuations, implying enhanced dynamic motion upon binding.

Figure 11Graphical representation of (a) RMSD, (b) RMSF, (c) RoG, and (d) SASA values across C*α* of *Mu*DHFR‐Apo (black), *Mu*DHFR‐P218 complex (red), *Mu*DHFR–methotrexate complex (green), *Mu*DHFR–trimethoprim complex (blue), *Mu*DHFR–3‐glucosylmaclurin complex (cyano), *Mu*DHFR–6‐O‐galloylglucose complex (magenta), *Mu*DHFR–ellagic acid complex (yellow), *Mu*DHFR–kaempferol‐3‐O‐rutinoside complex (dark yellow), *Mu*DHFR–neochlorogenic acid complex (navy), and *Mu*DHFR*–*procyanidin complex (purple) over 300‐ns MD simulations.

**Figure:**
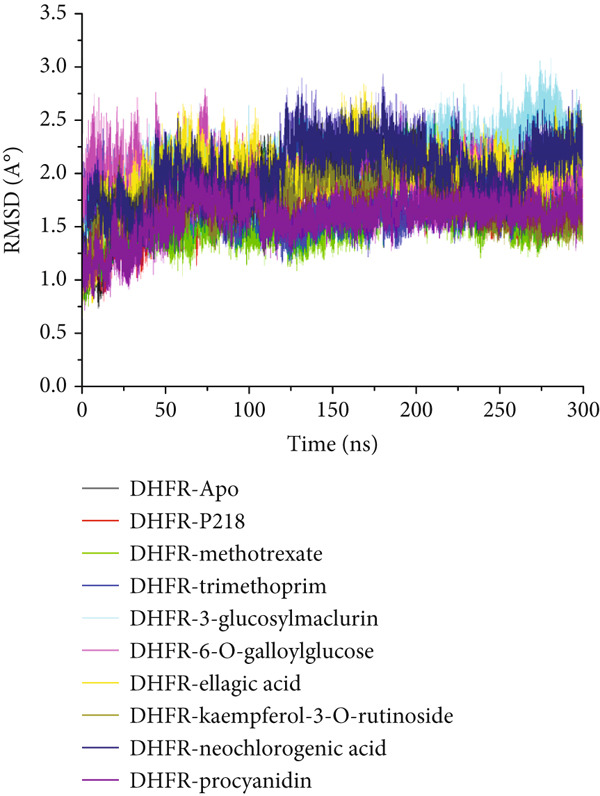
(a)

**Figure:**
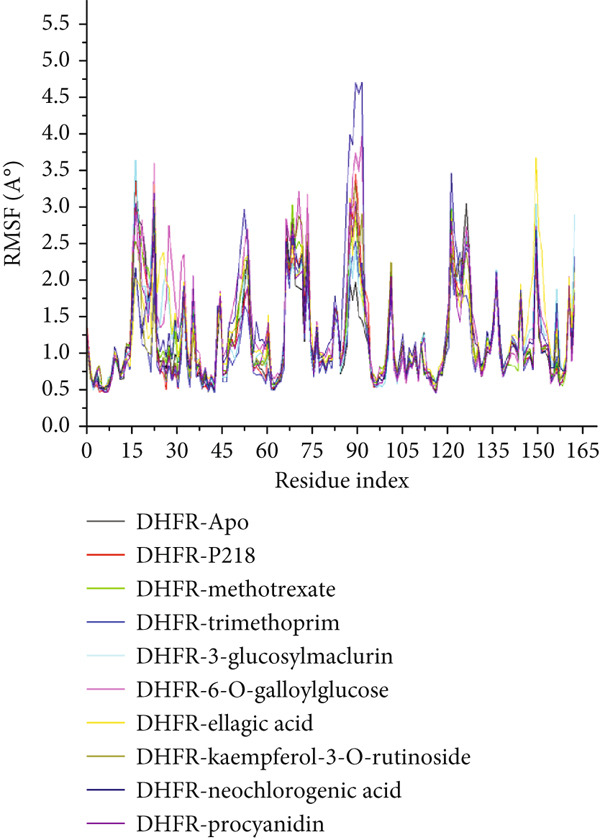
(b)

**Figure:**
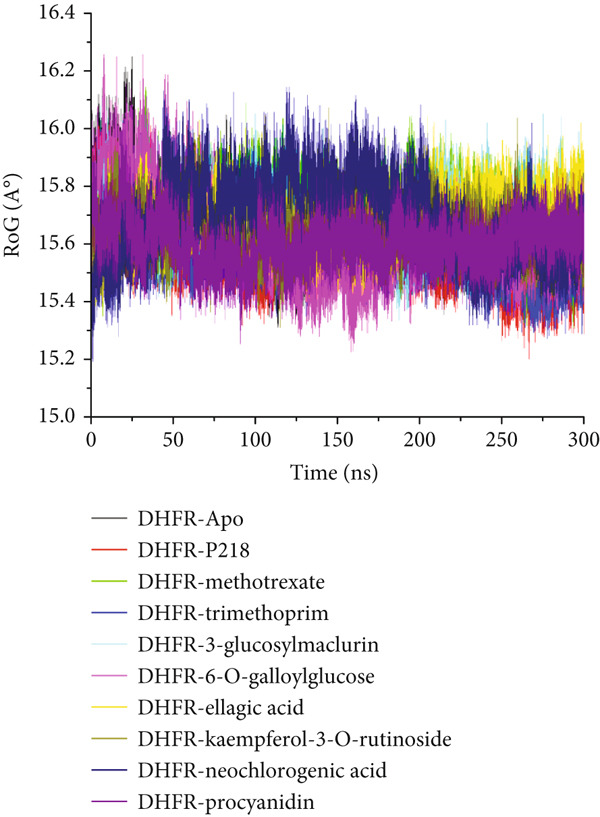
(c)

**Figure:**
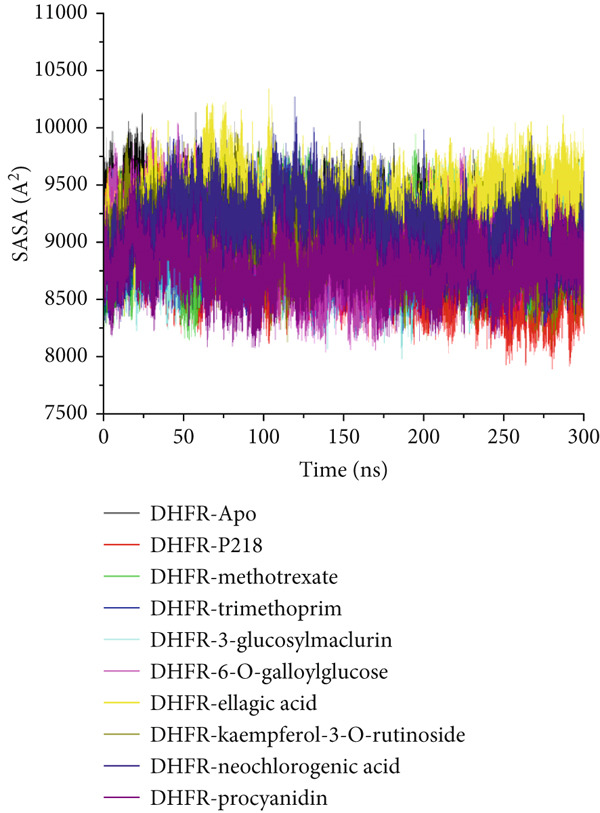
(d)

**Table 8:** RMSD, RMSF, RoG, and SASA profiles of Apo *Mu*DHFR and when bound to ligands.

**Systems**	**Estimated averages (Å)**			
**Ligand**	**RMSD**	**RMSF**	**RoG**	**SASA**
Apo	1.92	1.17	15.66	9207.74
P218	1.73	1.23	15.58	8724.92
Kaempferol‐3‐O‐rutinoside	1.75	1.22	15.65	8922.53
Neochlorogenic acid	2.01	1.39	15.70	9104.69
3‐Glucosylmaclurin	2.08	1.26	15.69	8947.63
Methotrexate	1.57	1.24	15.70	9030.92
Procyanidin	1.58	1.20	15.61	8764.72
Trimethoprim	1.72	1.08	15.58	9010.86
6‐O‐Galloylglucose	1.99	1.40	15.63	8967.04
Ellagic acid	2.00	1.34	15.70	9318.51

To better understand protein compaction, we evaluated the RoG throughout the MD simulations. RoG measures were computed as mass‐weighted root mean square distances from the complex’s center of mass. As displayed in Figure 11c and Table 8, ligands such as P218, kaempferol‐3‐O‐rutinoside, procyanidin, trimethoprim, and 6‐O‐galloylglucose promoted a more compact protein architecture, indicating enhanced rigidity. In contrast, methotrexate, neochlorogenic acid, 3‐glucosylmaclurin, and ellagic acid induced more extended or less compact conformations.

SASA calculations provided additional insight into protein surface exposure. These values reflect the extent to which amino acid residues are exposed to solvent, influencing hydrophobic interactions and folding. A decrease in SASA generally correlates with a more folded and stable protein structure [81]. Most ligand‐bound complexes—especially those with P218, kaempferol‐3‐O‐rutinoside, neochlorogenic acid, 3‐glucosylmaclurin, methotrexate, procyanidin, trimethoprim, and 6‐O‐galloylglucose—exhibited reduced SASA values relative to the Apo state, suggesting decreased solvent exposure and increased folding. Notably, ellagic acid was the exception, causing an increase in SASA, which may indicate structural loosening or partial unfolding (Figure 11d and Table 8).

#### 3.8.4. Investigation of Motional Dynamics via PCA

**Figure 12:**
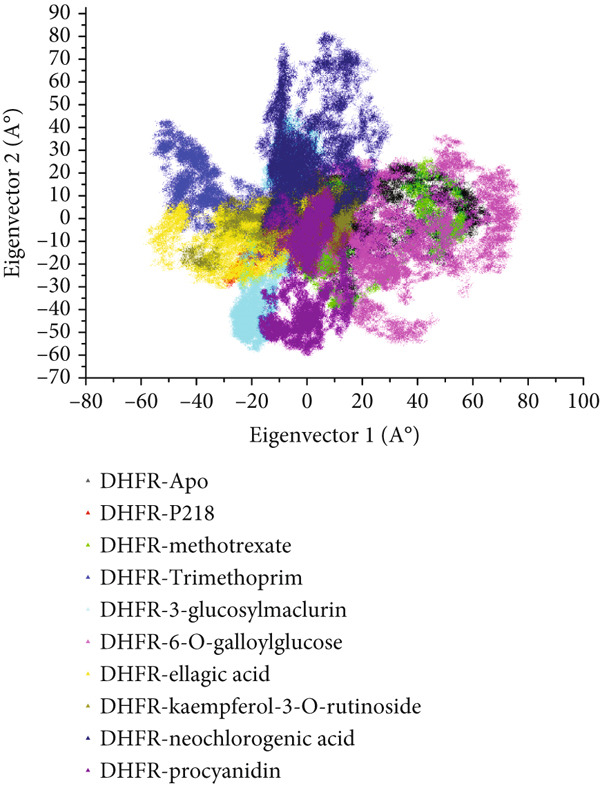
Graphical representation of principal component analysis (PCA) for *Mu*DHFR–Apo (black), *Mu*DHFR–P218 complex (red), *Mu*DHFR–methotrexate complex (green), *Mu*DHFR–trimethoprim complex (blue), *Mu*DHFR–3‐glucosylmaclurin complex (cyano), *Mu*DHFR–6‐O‐galloylglucose complex (magenta), *Mu*DHFR–ellagic acid complex (yellow), *Mu*DHFR–kaempferol‐3‐O‐rutinoside complex (dark yellow), *Mu*DHFR–neochlorogenic acid complex (navy), and *Mu*DHFR–procyanidin complex (purple) over 300‐ns MD simulations.

To explore the global conformational changes influenced by ligand binding, PCA was performed using the first two principal eigenvectors (EV_1_ and EV_2_). This method captures the most significant movements in the protein’s dynamic behavior. Two‐dimensional PCA scatter plots (Figure 12) illustrated the distinct motion profiles of *Mu*DHFR in its unbound form and when complexed with the ligands. The Apo form and the neochlorogenic acid complex displayed a balance of positively and negatively correlated motions, indicating diverse residue dynamics. In contrast, complexes with methotrexate, 6‐O‐galloylglucose, and procyanidin were characterized by more positively correlated, constrained motions, implying limited fluctuation. Conversely, systems involving P218, trimethoprim, 3‐glucosylmaclurin, ellagic acid, and kaempferol‐3‐O‐rutinoside showed dominant anticorrelated motions, with more pronounced fluctuations and widespread residue movement.

## 4. Conclusion

This *in silico* investigation, reinforced by MD simulations, has successfully identified promising phytochemical inhibitors of *Mu*DHFR from medicinal plants used in the traditional treatment of BU in West Africa. Our findings reveal that kaempferol‐3‐O‐rutinoside and neochlorogenic acid are the most compelling lead candidates. They exhibit binding free energies superior to the standard antifolate drugs methotrexate and trimethoprim; their interactions are characterized by sustained dynamic stability and the formation of key interactions with critical arginine residues in the *Mu*DHFR active site. The favorable ADMET profiles of these compounds, particularly their predicted lack of skin sensitization, further support their suitability for development as topical therapeutic agents. This work provides a strong computational foundation and a clear direction for the next phase of drug discovery. We strongly advocate for the experimental validation of kaempferol‐3‐O‐rutinoside and neochlorogenic acid to confirm their inhibitory activity against *Mu*DHFR and their efficacy against *M. ulcerans*. The development of a multicomponent topical formulation comprising different proportions of these promising natural products could lead to novel and accessible therapeutic options for BU treatment.

## Conflicts of Interest

The authors declare no conflict of interest.

## Funding

No funding was received for this manuscript.

## Supporting information


**Supporting Information** Additional supporting information can be found online in the Supporting Information section. Supporting information, including detailed tables and additional data supporting the findings of this study, are provided as part of the online supporting information. Table S1: Phytochemicals reported from the four West African medicinal plants used in the herbal formulations clinically evaluated by Trébissou et al. [5]. Table S2: Molecular docking and Prime MM‐GBSA results.

## Data Availability

The data that support the findings of this study are available from the corresponding author upon reasonable request.
