# Supplementary material for: In Silico Investigation of Phytochemicals From Clinically Tested Herbal Extracts as Potential Dihydrofolate Reductase Inhibitors for Buruli Ulcer
Source: Biomed Res Int. 2025 Sep 3;2025:4196295. doi: 10.1155/bmri/4196295 (PMC12407312; doi:10.1155/bmri/4196295)
Supplement: Supplementary file 1 — Supporting Information Additional supporting information can be found online in the Supporting Information section. Supporting information, including detailed tables and additional data supporting the findings of this study, are provided as part of the online supporting information. Table S1: Phytochemicals reported from the four West African medicinal plants used in the herbal formulations clinically evaluated by Trébissou et al. [5]. Table S2: Molecular docking and Prime MM‐GBSA results. [file BMRI-2025-4196295-s001.docx]

**Supplementary Information**

***In Silico* Investigation of Phytochemicals from Clinically Tested Herbal Extracts as Potential Dihydrofolate Reductase Inhibitors for** **Buruli Ulcer**

Alkhair Adam Khalil Mohamed^1^, Tamekloe Woasiedem^1^, Philip Collins Appiah-Ofori^1^, Esraa Mohammed Abdulmahmoud Adam^1^**,** Samuel Asiamah-Obiri^2^, Lawrence Sheringham Borquaye^3,4^, Christian K. Adokoh^5^, Ghazi Elamin^6^, Isaac Asiamah^2, *^

*^1^Department of Biomedical Sciences, School of Allied Health Sciences, College of Health and Allied Sciences, University of Cape Coast, Cape Coast, Ghana*

*^2^Department of Chemistry, School of Physical Sciences, College of Agriculture and Natural Sciences, University of Cape Coast, Cape Coast, Ghana*

*^3^Department of Chemistry, Kwame Nkrumah University of Science and Technology, Kumasi, Ghana*

*^4^Central Laboratory, Kwame Nkrumah University of Science and Technology, Kumasi, Ghana*

*^5^Department of Forensic Sciences, School of Biological Sciences, College of Agriculture and Natural Sciences University of Cape Coast, Cape Coast, Ghana*

*^6^Department of Pharmaceutical Chemistry, College of Pharmacy, Karary University, Khartoum, Sudan*

^*^Corresponding Author. Email: [iasiamah1@ucc.edu.gh](mailto:iasiamah1@ucc.edu.gh)

**Table S1:** Phytochemicals reported from the four West African medicinal plants used in the herbal formulations clinically evaluated by Trébissou et al. (2014).

| **No.** | **PubChem CID** | **Compound’s name** | **Origin** | **Reference** |
| --- | --- | --- | --- | --- |
| **1** | 54670067 | Ascorbic acid | *Carica papaya* | (Airaodion et al., 2020) |
| **2** | 222284 | Beta-sitosterol | *Carica papaya* | (Khaw et al., 2020) |
| **3** | 173183 | Campesterol | *Carica papaya* | (Sani et al., 2020) |
| **4** | 442630 | Carpaine | *Carica papaya* | (Pertiwi et al., 2019) |
| **5** | 305 | Choline | *Carica papaya* | (Gunde & Amnerkar, 2016) |
| **6** | 2116 | DL-alpha-Tocopherol | *Carica papaya* | (Pambhar et al., 2022) |
| **7** | 445858 | Ferulic acid | *Carica papaya* | (Kong et al., 2021) |
| **8** | 9548605 | Glucotropaeolin | *Carica papaya* | (Castro-Vargas et al., 2016) |
| **9** | 12410 | Hentriacontane | *Carica papaya* | (Haber et al., 2022) |
| **10** | 6537198 | Indolylglucosinolate | *Carica papaya* | (Gonza et al., 2011) |
| **11** | 114829 | Liquiritigenin | *Carica papaya* | (Muntholib et al., 2020) |
| **12** | 446925 | Lycopene | *Carica papaya* | (Lara-Abia et al., 2021) |
| **13** | 1292 | Mandelic acid | *Carica papaya* | (Sani et al., 2020) |
| **14** | 107202 | Methoxyphenylacetic acid | *Carica papaya* | (Gonçalves Rodrigues et al., 2019) |
| **15** | 445154 | Resveratrol | *Carica papaya* | (Feng et al., 2022) |
| **16** | 493570 | Riboflavin | *Carica papaya* | (Chinasa et al., 2022) |
| **17** | 338 | Salicylic acid | *Carica papaya* | (Ntakoulas et al., 2023) |
| **18** | 5280794 | Stigmasterol | *Carica papaya* | (Haber et al., 2022) |
| **19** | 5280343 | Quercetin | *Carica papaya Mangifera indica* | (Kong et al., 2021) (Quintana et al., 2021) |
| **20** | 5280450 | Linoleic acid | *Carica papaya* | (Haber et al., 2022) |
| **21** | 5280934 | Linolenic acid | *Carica papaya* | (Haber et al., 2022) |
| **22** | 12303902 | (-)-alpha-Copaene | *Mangifera indica* | (Mehmood et al., 2024) |
| **23** | 131751910 | (3b,22S,24E)-3,22-Dihydroxycycloart-24-en-26-oic acid | *Mangifera indica* | (Mehmood et al., 2024) |
| **24** | 14314551 | (3beta,24xi)-Cycloart-25-ene-3,24,27-triol | *Mangifera indica* | (Mehmood et al., 2024) |
| **25** | 5270670 | Cycloartane-3,24,25-triol | *Mangifera indica* | (Mehmood et al., 2024) |
| **26** | 2723720 | (5R)-2,6,6-trimethylbicyclo[3.1.1]hept-2-ene | *Mangifera indica* | (Mehmood et al., 2024) |
| **27** | 1203 | 2-(3,4-Dihydroxyphenyl)chroman-3,5,7-triol | *Mangifera indica* | (Quintana et al., 2021) |
| **28** | 14529 | 2-(4-Methylphenyl)propan-2-ol | *Mangifera indica* | (Mehmood et al., 2024) |
| **29** | 79983 | 2,5-Di-tert-butylphenol | *Mangifera indica* | (Mehmood et al., 2024) |
| **30** | 709 | 3-(4-Hydroxy-3-methoxyphenyl)prop-2-enoic acid | *Mangifera indica* | (Mehmood et al., 2024) |
| **31** | 72 | 3,4-Dihydroxybenzoic acid | *Mangifera indica* | (Mehmood et al., 2024) |
| **32** | 131752926 | 3beta-Cycloartane-3,29-diol | *Mangifera indica* | (Mehmood et al., 2024) |
| **33** | 75169766 | 3-Glucosyl-2,3',4,4',6-pentahydroxybenzophenone | *Mangifera indica* | (Mehmood et al., 2024) |
| **34** | 135 | 4-Hydroxybenzoic acid | *Mangifera indica* | (Mehmood et al., 2024) |
| **35** | 11230 | 4-terpineol, (+/-)- | *Mangifera indica* | (Mehmood et al., 2024) |
| **36** | 128839 | 6-o-galloylglucose | *Mangifera indica* | (Quintana et al., 2021) |
| **37** | 131801655 | 9-cis-Lutein | *Mangifera indica* | (Chen et al., 2007) |
| **38** | 12306048 | Alpha-Cadinene, (+)- | *Mangifera indica* | (Mehmood et al., 2024) |
| **39** | 80048 | Alpha-Elemene | *Mangifera indica* | (Mehmood et al., 2024) |
| **40** | 92762 | Alpha-Eudesmol | *Mangifera indica* | (Mehmood et al., 2024) |
| **41** | 5317844 | Alpha-Guaiene | *Mangifera indica* | (Mehmood et al., 2024) |
| **42** | 578929 | Alpha-Panasinsene | *Mangifera indica* | (Mehmood et al., 2024) |
| **43** | 10856614 | Alpha-Selinene | *Mangifera indica* | (Mehmood et al., 2024) |
| **44** | 5281600 | Amentoflavone | *Mangifera indica* | (Mehmood et al., 2024) |
| **45** | 126843388 | Apigenin 7-O-diglucuronide | *Mangifera indica* | (Quintana et al., 2021) |
| **46** | 5280704 | Apigetrin | *Mangifera indica* | (Quintana et al., 2021) |
| **47** | 2259 | Aurintricarboxylic acid | *Mangifera indica* | (Quintana et al., 2021) |
| **48** | 124021 | Beta-Glucogallin | *Mangifera indica* | (Quintana et al., 2021) |
| **49** | 689043 | Caffeic acid | *Mangifera indica* | (Mehmood et al., 2024) |
| **50** | 2537 | Camphor | *Mangifera indica* | (Mehmood et al., 2024) |
| **51** | 10374 | Chlorazanil | *Mangifera indica* | (Quintana et al., 2021) |
| **52** | 9064 | Cianidanol | *Mangifera indica* | (Quintana et al., 2021) |
| **53** | 16213731 | Cid 16213731 | *Mangifera indica* | (Mehmood et al., 2024) |
| **54** | 444539 | Cinnamic acid | *Mangifera indica* | (Mehmood et al., 2024) |
| **55** | 3084311 | Delta-Cadinol | *Mangifera indica* | (Mehmood et al., 2024) |
| **56** | 92138 | Elemol | *Mangifera indica* | (Mehmood et al., 2024) |
| **57** | 5281855 | Ellagic acid | *Mangifera indica* | (Quintana et al., 2021) |
| **58** | 65064 | Epigallocatechin gallate | *Mangifera indica* | (Mehmood et al., 2024) |
| **59** | 12309744 | Eremophilene | *Mangifera indica* | (Mehmood et al., 2024) |
| **60** | 7362 | Furfural | *Mangifera indica* | (Mehmood et al., 2024) |
| **61** | 521334 | Gamma-Selinene | *Mangifera indica* | (Mehmood et al., 2024) |
| **62** | 5281858 | Ginkgolic acid | *Mangifera indica* | (Quintana et al., 2021) |
| **63** | 12304985 | Globulol | *Mangifera indica* | (Mehmood et al., 2024) |
| **64** | 41774 | Glucobay | *Mangifera indica* | (Mehmood et al., 2024) |
| **65** | 785 | Hydroquinone | *Mangifera indica* | (Mehmood et al., 2024) |
| **66** | 3663 | Hypericin | *Mangifera indica* | (Mehmood et al., 2024) |
| **67** | 5281643 | Hyperoside | *Mangifera indica* | (Quintana et al., 2021) |
| **68** | 11311158 | Iriflophenone | *Mangifera indica* | (Mehmood et al., 2024) |
| **69** | 184358 | Iriflophenone 3-C-glucoside | *Mangifera indica* | (Mehmood et al., 2024) |
| **70** | 5318597 | Isomangiferin | *Mangifera indica* | (Mehmood et al., 2024) |
| **71** | 10813969 | Isoquercitin | *Mangifera indica* | (Quintana et al., 2021) |
| **72** | 44258317 | Isoswertisin | *Mangifera indica* | (Quintana et al., 2021) |
| **73** | 162350 | Isovitexin | *Mangifera indica* | (Mehmood et al., 2024) |
| **74** | 5280863 | Kaempferol | *Mangifera indica* | (Quintana et al., 2021) |
| **75** | 259846 | Lupeol | *Mangifera indica* | (Mehmood et al., 2024) |
| **76** | 5280637 | Luteolin 7-O-glucoside | *Mangifera indica* | (Kumar et al., 2021) |
| **77** | 68213 | Maclurin | *Mangifera indica* | (Mehmood et al., 2024) |
| **78** | 5281647 | Mangiferin | *Mangifera indica* | (Quintana et al., 2021) |
| **79** | 14034474 | Mangiferonic acid | *Mangifera indica* | (Mehmood et al., 2024) |
| **80** | 181681 | Medioresinol | *Mangifera indica* | (Quintana et al., 2021) |
| **81** | 7428 | Methyl gallate | *Mangifera indica* | (Mehmood et al., 2024) |
| **82** | 5281672 | Myricetin | *Mangifera indica* | (Quintana et al., 2021) |
| **83** | 938 | Nicotinic acid | *Mangifera indica* | (Mehmood et al., 2024) |
| **84** | 5281656 | Norathyriol | *Mangifera indica* | (Mehmood et al., 2024) |
| **85** | 110745 | Palustrol | *Mangifera indica* | (Mehmood et al., 2024) |
| **86** | 637542 | P-Coumaric acid | *Mangifera indica* | (Mehmood et al., 2024) |
| **87** | 65238 | Pentagalloylglucose | *Mangifera indica* | (Quintana et al., 2021) |
| **88** | 11468 | P-Menth-3-en-1-ol | *Mangifera indica* | (Mehmood et al., 2024) |
| **89** | 107876 | Procyanidin | *Mangifera indica* | (Quintana et al., 2021) |
| **90** | 1057 | Pyrogallol | *Mangifera indica* | (Mehmood et al., 2024) |
| **91** | 25203368 | Quercetin-3-glucoside | *Mangifera indica* | (Quintana et al., 2021) |
| **92** | 13784485 | Quercetin-3-O-deoxyhexosyl(1-2) pentoside | *Mangifera indica* | (Mehmood et al., 2024) |
| **93** | 5280459 | Quercitrin | *Mangifera indica* | (Mehmood et al., 2024) |
| **94** | 5274585 | Querciturone | *Mangifera indica* | (Mehmood et al., 2024) |
| **95** | 445354 | Retinol | *Mangifera indica* | (Mehmood et al., 2024) |
| **96** | 5281792 | Rosmarinic acid | *Mangifera indica* | (Quintana et al., 2021) |
| **97** | 439533 | Taxifolin | *Mangifera indica* | (Mehmood et al., 2024) |
| **98** | 11463 | Terpinolene | *Mangifera indica* | (Mehmood et al., 2024) |
| **99** | 442988 | Theogallin | *Mangifera indica* | (Mehmood et al., 2024) |
| **100** | 29986831 | Trans-Resveratrol 3,5-disulfate | *Mangifera indica* | (Quintana et al., 2021) |
| **101** | 5281702 | Tricin | *Mangifera indica* | (Mehmood et al., 2024) |
| **102** | 8468 | Vanillic acid | *Mangifera indica* | (Mehmood et al., 2024) |
| **103** | 11996452 | Viridiflorol | *Mangifera indica* | (Mehmood et al., 2024) |
| **104** | 6042 | Vitamin B1 | *Mangifera indica* | (Mehmood et al., 2024) |
| **105** | 5280441 | Vitexin | *Mangifera indica* | (Mehmood et al., 2024) |
| **106** | 12112747 | Luteoxanthin | *Mangifera indica* | (Kanupriya et al., 2016) |
| **107** | 5280899 | Zeaxanthin | *Nicotiana tabacum* | (Quintana et al., 2021) |
| **108** | 11968014 | Scopolamine | *Nicotiana tabacum* | (Zhang et al., 2024) |
| **109** | 547 | 3,4-Dihydroxyphenylacetic acid | *Nicotiana tabacum* | (Zou et al., 2021) |
| **110** | 10133609 | 3-O-(E)-Feruloylquinic acid | *Nicotiana tabacum* | (Zou et al., 2021) |
| **111** | 5281766 | 4-p-Coumaroylquinic acid | *Nicotiana tabacum* | (Zou et al., 2021) |
| **112** | 1794427 | Chlorogenic acid | *Nicotiana tabacum* | (Zou et al., 2021) |
| **113** | 6167 | Colchicine | *Nicotiana tabacum* | (Zou et al., 2021) |
| **114** | 9798666 | Cryptochlorogenic acid | *Nicotiana tabacum* | (Zou et al., 2021) |
| **115** | 5366074 | Damascenone | *Nicotiana tabacum* | (Zhang et al., 2024) |
| **116** | 5318767 | Kaempferol-3-O-rutinoside | *Nicotiana tabacum* | (Zou et al., 2021) |
| **117** | 11005 | Myristic acid | *Nicotiana tabacum* | (Zou et al., 2021) |
| **118** | 5280633 | Neochlorogenic acid | *Nicotiana tabacum* | (Zou et al., 2021) |
| **119** | 10446 | Neophytadiene | *Nicotiana tabacum* | (Zhang et al., 2024) |
| **120** | 89594 | Nicotine | *Nicotiana tabacum* | (Zou et al., 2021) |
| **121** | 445639 | Oleic acid | *Nicotiana tabacum* | (Zou et al., 2021) |
| **122** | 5477212 | Solanesol | *Nicotiana tabacum* | (Zou et al., 2021) |
| **123** | 6451337 | Solanone | *Nicotiana tabacum* | (Zhang et al., 2024) |
| **124** | 5280805 | Rutin | *Nicotiana tabacum* | (Kumaree et al., 2023) |
| **125** | 985 | Palmitic acid | *Nicotiana tabacum* | (Zou et al., 2021) |
| **126** | 12306055 | (-)-delta-Cadinene | *Solanum rugosum* | (Kouao et al., 2021) |
| **127** | 6432404 | (+)-gamma-Cadinene | *Solanum rugosum* | (Kouao et al., 2021) |
| **128** | 6431302 | Alpha-Cadinol | *Solanum rugosum* | (Kouao et al., 2021) |
| **129** | 6429032 | Alpha-Elemol | *Solanum rugosum* | (Kouao et al., 2021) |
| **130** | 92762 | Alpha-Eudesmol | *Solanum rugosum* | (Kouao et al., 2021) |
| **131** | 17100 | Alpha-terpineol | *Solanum rugosum* | (Kouao et al., 2021) |
| **132** | 1742210 | Beta-CARYOPHYLLENE OXIDE | *Solanum rugosum* | (Kouao et al., 2021) |
| **133** | 6918391 | Beta-elemene | *Solanum rugosum* | (Kouao et al., 2021) |
| **134** | 91457 | Beta-EUDESMOL | *Solanum rugosum* | (Kouao et al., 2021) |
| **135** | 638014 | Beta-Ionone | *Solanum rugosum* | (Kouao et al., 2021) |
| **136** | 13894537 | Bicyclogermacrene | *Solanum rugosum* | (Kouao et al., 2021) |
| **137** | 90785 | Bulnesol | *Solanum rugosum* | (Kouao et al., 2021) |
| **138** | 5281515 | Caryophyllene | *Solanum rugosum* | (Kouao et al., 2021) |
| **139** | 3037997 | Corynantheine | *Solanum rugosum* | (Calixte et al., 2021) |
| **140** | 92766 | Corynanthine | *Solanum rugosum* | (Calixte et al., 2021) |
| **141** | 5366074 | Damascenone | *Solanum rugosum* | (Kouao et al., 2021) |
| **142** | 6432005 | Gamma-EUDESMOL | *Solanum rugosum* | (Kouao et al., 2021) |
| **143** | 637566 | Geraniol | *Solanum rugosum* | (Kouao et al., 2021) |
| **144** | 5317570 | Germacrene D | *Solanum rugosum* | (Kouao et al., 2021) |
| **145** | 227829 | Guaiol | *Solanum rugosum* | (Kouao et al., 2021) |
| **146** | 5281520 | Humulene | *Solanum rugosum* | (Kouao et al., 2021) |
| **147** | 6549 | Linalool | *Solanum rugosum* | (Kouao et al., 2021) |
| **148** | 72378 | Lycorine | *Solanum rugosum* | (Calixte et al., 2021) |
| **149** | 5280435 | Phytol | *Solanum rugosum* | (Kouao et al., 2021) |
| **150** | 5281408 | Rhynchophylline | *Solanum rugosum* | (Calixte et al., 2021) |
| **151** | 61041 | Safranal | *Solanum rugosum* | (Kouao et al., 2021) |
| **152** | 9546749 | 1-Stearoyl-2-linoleoyl-sn-glycero-3-phosphoethanolamine | *Solanum torvum* | (Soorya et al., 2017) |
| **153** | 129837279 | 2,3,4-trimethyltriacontane | *Solanum torvum* | (Vaithilingam et al., 2021) |
| **154** | 12304761 | 2-[4-(16-Amino-6-hydroxy-trimethyl-5-oxapentacyclo[1]icosan-6-yl)-2-methylbutoxy]-6-(hydroxymethyl)oxanetriol | *Solanum torvum* | (Vaithilingam et al., 2021) |
| **155** | 628858 | 3,3-Bis(4-hydroxy-2,5-dimethylphenyl)isobenzofuran-1(3H)-one | *Solanum torvum* | (Soorya et al., 2017) |
| **156** | 545660 | 3-tritriacontanone | *Solanum torvum* | (Vaithilingam et al., 2021) |
| **157** | 129847814 | 5-hexatriacontanone | *Solanum torvum* | (Vaithilingam et al., 2021) |
| **158** | 131753159 | Torvoside E | *Solanum torvum* | (Vaithilingam et al., 2021) |
| **159** | 5365703 | Ambrettolide | *Solanum torvum* | (Soorya et al., 2017) |
| **160** | 22212741 | Cholesta-5,7,9(11)-trien-3-ol,4,4-dimethyl-, (3beta)- | *Solanum torvum* | (Soorya et al., 2017) |
| **161** | 10047336 | Cholesta-5,7-dien-3-ol, 4,4-dimethyl-, (3beta)- | *Solanum torvum* | (Soorya et al., 2017) |
| **162** | 22295249 | Cholestan-26-oic acid, 3,7,12,24-tetrakis(acetyloxy)-, methyl ester | *Solanum torvum* | (Soorya et al., 2017) |
| **163** | 12366 | Ethyl palmitate | *Solanum torvum* | (Soorya et al., 2017) |
| **164** | 22212804 | Lanosta-7,9(11),20-triene-3beta,18-diol, diacetate | *Solanum torvum* | (Soorya et al., 2017) |
| **165** | 5364509 | Methyl oleate | *Solanum torvum* | (Soorya et al., 2017) |
| **166** | 70697823 | Chebi:66258 | *Solanum torvum* | (Vaithilingam et al., 2021) |
| **167** | 13889197 | Spirostane-3,6-dione | *Solanum torvum* | (Vaithilingam et al., 2021) |
| **168** | 5321987 | Torvanol A | *Solanum torvum* | (Vaithilingam et al., 2021) |
| **169** | 131751074 | Torvonin A | *Solanum torvum* | (Vaithilingam et al., 2021) |
| **170** | 131753157 | Torvoside A | *Solanum torvum* | (Vaithilingam et al., 2021) |
| **171** | 131753160 | Torvoside F | *Solanum torvum* | (Vaithilingam et al., 2021) |
| **172** | 70697829 | Torvoside H | *Solanum torvum* | (Vaithilingam et al., 2021) |

**Table S2:** Molecular Docking and Prime MM-GBSA Results

| No. | Title | Docking score | MMGBSA ΔG_Bind_  kcal/mol |
| --- | --- | --- | --- |
| 1 | Tetra-o-galloylglucose | -13.599 | -44.68 |
| 2 | P218 | -11.875 | -70.5 |
| 3 | Rutin | -11.8 | -48.05 |
| 4 | Kaempferol-3-O-rutinoside | -11.23 | -53.2 |
| 5 | Procyanidin | -11.137 | -54.17 |
| 6 | 3-Glucosyl-2,3',4,4',6-pentahydroxybenzophenone | -10.616 | -56.03 |
| 7 | Isomangiferin | -10.46 | -39.87 |
| 8 | quercetin-3-O-deoxyhexosyl(1-2)pentoside | -10.393 | -32.14 |
| 9 | Luteolin 7-O-glucoside | -10.084 | -52.4 |
| 10 | Methotrexate | -10.04 | -58.63 |
| 11 | 6-O-Galloylglucose | -9.988 | -64.06 |
| 12 | Vitexin | -9.797 | -42.98 |
| 13 | Iriflophenone 3-C-glucoside | -9.652 | -39.24 |
| 14 | Apigetrin | -9.646 | -42.03 |
| 15 | Hyperoside | -9.314 | -41.72 |
| 16 | Trimethoprim | -9.291 | -64.39 |
| 17 | Mangiferin | -9.289 | -44.92 |
| 18 | Riboflavin | -9.269 | -37.65 |
| 19 | 6-Methoxy-7,9,13-trimethyl-6-[3-methyl-4-[3,4,5-trihydroxy-6-(hydroxymethyl)oxan-2-yl]oxybutyl]-19-(3,4,5-trihydroxy-6-methyloxan-2-yl)oxy-5-oxapentacyclo[10.8.0.02,9.04,8.013,18]icosan-16-one | -9.19 | -42.16 |
| 20 | Quercetin-3-glucoside | -9.174 | -39.14 |
| 21 | Neochlorogenic acid | -9.055 | -52.57 |
| 22 | Myricetin | -8.881 | -47.09 |
| 23 | Isovitexin | -8.855 | -50.3 |
| 24 | Epigallocatechin Gallate | -8.608 | -50.46 |
| 25 | beta-Glucogallin | -8.316 | -40.35 |
| 26 | Ellagic Acid | -8.159 | -53.31 |
| 27 | Quercetin | -7.952 | -46.17 |
| 28 | Quercitrin | -7.911 | -44.02 |
| 29 | Maclurin | -7.894 | -27.02 |
| 30 | Isoswertisin | -7.8 | -41.26 |
| 31 | 2-(3,4-Dihydroxyphenyl)chroman-3,5,7-triol | -7.784 | -52.53 |
| 32 | rel-potassium (3R,4S)-3-{5-[(E)-2-carboxyethenyl]-2-hydroxy-3-methoxyphenyl}-6-methoxy-3,4-dihydro-2H-chromen-4-yl sulfate | -7.611 | -49.74 |
| 33 | Ferulic acid | -7.565 | -30.99 |
| 34 | 3-(4-Hydroxy-3-methoxyphenyl)prop-2-enoic acid | -7.565 | -30.99 |
| 35 | Kaempferol | -7.463 | -30.79 |
| 36 | Chlorazanil | -7.443 | -40.89 |
| 37 | (3R,6R)-6-[(1S,3R,6S,8R,11S,12S,15R,16R)-6-hydroxy-7,7,12,16-tetramethyl-15-pentacyclo[9.7.0.01,3.03,8.012,16]octadecanyl]-2-methylheptane-2,3-diol | -7.321 | -9.73 |
| 38 | Isoquercitin | -7.02 | -27.96 |
| 39 | Cianidanol | -6.926 | -43.06 |
| 40 | Amentoflavone | -6.79 | -54.43 |
| 41 | Methyl gallate | -6.625 | -28.68 |
| 42 | Medioresinol | -6.565 | -57.36 |
| 43 | (3beta,24xi)-Cycloart-25-ene-3,24,27-triol | -6.538 | -43.14 |
| 44 | 3beta-Cycloartane-3,29-diol | -6.222 | -22.96 |
| 45 | Ambrettolide | -6.19 | -39.6 |
| 46 | Nicotine | -6.157 | -29.36 |
| 47 | 2,5-Di-tert-butylphenol | -6.126 | -35.59 |
| 48 | Norathyriol | -6.107 | -33.84 |
| 49 | Palustrol | -6.078 | -23.71 |
| 50 | Iriflophenone | -6.037 | -33.62 |
| 51 | Myristic Acid | -5.984 | -31.74 |
| 52 | Resveratrol | -5.868 | -42.35 |
| 53 | 3,3-Bis(4-hydroxy-2,5-dimethylphenyl)isobenzofuran-1(3H)-one | -5.833 | -46.08 |
| 54 | Bicyclogermacrene | -5.778 | -25.92 |
| 55 | Tricin | -5.72 | -41.21 |
| 56 | Pyrogallol | -5.696 | -29.02 |
| 57 | Retinol | -5.617 | -39.51 |
| 58 | [(1R,2S,4S,5S)-9-methyl-3-oxa-9-azatricyclo[3.3.1.02,4]nonan-7-yl] (2S)-3-hydroxy-2-phenylpropanoate | -5.554 | -49.08 |
| 59 | Vanillic Acid | -5.549 | -27.85 |
| 60 | Caryophyllene | -5.505 | -25.86 |
| 61 | Liquiritigenin | -5.461 | -38.58 |
| 62 | beta-CARYOPHYLLENE OXIDE | -5.42 | -32.4 |
| 63 | Lupeol | -5.396 | -1.69 |
| 64 | Querciturone | -5.365 | -49.3 |
| 65 | Germacrene D | -5.301 | -28.47 |
| 66 | gamma-EUDESMOL | -5.282 | -29.9 |
| 67 | Colchicine | -5.281 | -51.48 |
| 68 | alpha-Cadinol #1 | -5.269 | -32.22 |
| 69 | Elemol | -5.214 | -28.37 |
| 70 | delta-Cadinol | -5.187 | -30.78 |
| 71 | Guaiol | -5.184 | -35.86 |
| 72 | alpha-Cadinene, (+)- | -5.178 | -30.34 |
| 73 | alpha-Elemol | -5.162 | -28.41 |
| 74 | beta-EUDESMOL | -5.148 | -33.25 |
| 75 | alpha-Eudesmol | -5.079 | -26.76 |
| 76 | Viridiflorol | -5.012 | -31.22 |
| 77 | CID 16213731 | -5 | -28.89 |
| 78 | Beta-Sitosterol | -4.927 | 9.08 |
| 79 | Eremophilene | -4.873 | -26.7 |
| 80 | Globulol | -4.767 | -35.52 |
| 81 | Solanone | -4.717 | -19.15 |
| 82 | gamma-Selinene | -4.693 | -28.45 |
| 83 | Torvonin A | -4.677 | -31.99 |
| 84 | alpha-Selinene | -4.645 | -32.35 |
| 85 | 4-Terpineol, (+/-)- | -4.634 | -32.08 |
| 86 | Safranal | -4.578 | -24.96 |
| 87 | Alpha-Terpineol | -4.552 | -32.31 |
| 88 | Stigmasterol | -4.548 | -36.07 |
| 89 | Campesterol | -4.53 | -40.88 |
| 90 | Spirostane-3,6-dione | -4.504 | -32.71 |
| 91 | (-)-delta-Cadinene | -4.473 | -26.37 |
| 92 | Bulnesol | -4.388 | -37.28 |
| 93 | p-Menth-3-en-1-ol | -4.379 | -24.37 |
| 94 | Cholesta-5,7-dien-3-ol, 4,4-dimethyl-, (3beta)- | -4.378 | -19.85 |
| 95 | (-)-alpha-Copaene | -4.339 | -32.41 |
| 96 | Beta-Elemene | -4.299 | -36.28 |
| 97 | Furfural | -4.299 | -19.72 |
| 98 | Damascenone | -4.289 | -16.6 |
| 99 | alpha-Guaiene | -4.261 | -29.02 |
| 100 | 2-(4-Methylphenyl)propan-2-ol | -4.185 | -29.99 |
| 101 | Humulene | -4.163 | -35.71 |
| 102 | alpha-Panasinsene | -4.157 | -27.83 |
| 103 | Cholesta-5,7,9(11)-trien-3-ol, 4,4-dimethyl-, (3beta)- | -4.137 | -25.88 |
| 104 | (+)-gamma-Cadinene | -4.068 | -25.68 |
| 105 | alpha-Elemene | -4.015 | -35.81 |
| 106 | Hydroquinone | -3.924 | -24.19 |
| 107 | Choline | -3.914 | -28.78 |
| 108 | beta-Ionone | -3.857 | -32.79 |
| 109 | Taxifolin | -3.807 | -45.6 |
| 110 | 3-O-(E)-Feruloylquinic acid | -3.758 | -49.45 |
| 111 | Hypericin | -3.724 | -30.92 |
| 112 | (5R)-2,6,6-trimethylbicyclo[3.1.1]hept-2-ene | -3.65 | -21.14 |
| 113 | Lycorine | -3.609 | -49.52 |
| 114 | Camphor | -3.459 | -16.45 |
| 115 | Linalool, (+/-)- | -3.457 | -28.18 |
| 116 | Phytol | -3.273 | -42.18 |
| 117 | (3b,22S,24E)-3,22-Dihydroxycycloart-24-en-26-oic acid | -3.208 | -8.78 |
| 118 | Methyl oleate | -3.147 | -45.53 |
| 119 | Terpinolene | -3.132 | -25.17 |
| 120 | Geraniol | -3.127 | -26.09 |
| 121 | 3,4-Dihydroxybenzoic acid | -3.101 | -35.88 |
| 122 | Rhynchophylline | -2.718 | -35.99 |
| 123 | Caffeic Acid | -2.305 | -34.41 |
| 124 | 3,4-Dihydroxyphenylacetic acid | -2.227 | -38.6 |
| 125 | Corynanthine | -2.197 | -44.79 |
| 126 | 4-Hydroxybenzoic acid | -2.155 | -34.8 |
| 127 | Cinnamic Acid | -2.02 | -28.01 |
| 128 | Ascorbic Acid | -2.015 | -30.67 |
| 129 | p-Coumaric acid | -1.902 | -32.79 |
| 130 | Mandelic Acid | -1.818 | -27.22 |
| 131 | Nicotinic acid | -1.65 | -28.1 |
| 132 | Oleic Acid | -1.439 | -52.89 |
| 133 | Rosmarinic acid | -1.295 | -56.48 |
| 134 | Corynantheine | -1.215 | -40.03 |
| 135 | Ethyl palmitate | -1.203 | -43.44 |
| 136 | 2-[4-(16-Amino-6-hydroxy-7,9,13-trimethyl-5-oxapentacyclo[10.8.0.02,9.04,8.013,18]icosan-6-yl)-2-methylbutoxy]-6-(hydroxymethyl)oxane-3,4,5-triol | -1.029 | -50 |
| 137 | Mangiferonic acid | -0.948 | -30.98 |
| 138 | Apigenin 7-O-diglucuronide | -0.806 | -40.14 |
| 139 | Ginkgolic acid | -0.656 | -40.4 |
| 140 | Methoxyphenylacetic acid | -0.516 | -32.08 |
| 141 | Linoleic Acid | -0.494 | -47.52 |
| 142 | Linolenic Acid | -0.405 | -47.11 |
| 143 | Solanesol | -0.285 | -30.24 |
| 144 | Salicylic Acid | -0.26 | -26.77 |
| 145 | Chlorogenic Acid | -0.087 | -47.54 |
| 146 | Theogallin | 0.009 | -31.93 |
| 147 | Cryptochlorogenic acid | 0.321 | -45.82 |
| 148 | Tetra-o-galloylglucose | 0.827 | -63 |
| 149 | Palmitic Acid | 1.052 | -44.65 |
| 150 | Neophytadiene | 1.07 | -50.64 |
| 151 | 4-p-Coumaroylquinic acid | 1.493 | -50.1 |
| 152 | Cholestan-26-oic acid, 3,7,12,24-tetrakis(acetyloxy)-, methyl ester, (3alpha,5beta,7alpha,12alpha)- | 4.181 | -49.06 |
| 153 | Glucotropaeolin | 4.733 | -63.1 |
| 154 | Indolylglucosinolate | 4.963 | -56.92 |
| 155 | Carpaine | 7.114 | -30.33 |
| 156 | Torvanol A | 7.27 | -57.24 |
| 157 | Aurintricarboxylic acid | 17.811 | -53.63 |
| 158 | trans-Resveratrol 3,5-disulfate | 19.825 | -51.47 |

**References**

Airaodion, A. I., Ekenjoku, J. A., Akaninyene, I. U., & Megwas, A. U. (2020). Antibacterial Potential of Ethanolic and Aqueous Extracts of Carica papaya Leaves. *Asian Journal of Biochemistry, Genetics and Molecular Biology*, *May*, 33-38. https://doi.org/10.9734/ajbgmb/2020/v3i330088

Calixte, B., N’dr, T.-A., Aminata, A.-O., Moriba, T., Jea, A., Ma, S., Lan, K., K, C., & Die, K. (2021). Phytochemical, Acute Toxicity and Tolerance Evaluation of Solanum rugosum (Solanaceae) on Skin and Eye. *Research Journal of Medicinal Plants*, *15*, 36-45. https://doi.org/10.3923/rjmp.2021.36.45

Castro-Vargas, H. I., Baumann, W., & Parada-Alfonso, F. (2016). Valorization of agroindustrial wastes: Identification by LC-MS and NMR of benzylglucosinolate from papaya (carica papaya L.) seeds, a protective agent against lipid oxidation in edible oils. *Electrophoresis*, *37*(13), 1930-1937. https://doi.org/10.1002/elps.201500499

Chen, J. P., Tai, C. Y., & Chen, B. H. (2007). Effects of different drying treatments on the stability of carotenoids in Taiwanese mango (Mangifera indica L. *Food Chemistry*, *100*(3), 1005-1010. https://doi.org/10.1016/j.foodchem.2005.10.056

Chinasa, O., Chukwunwike, U. E., Chinedu, O. O., Wisdom, N. E., & Emem, H. U. (2022). Antimicrobial Potentials of Carica Papaya Latex and Seed Extract. *IOSR Journal of Biotechnology and Biochemistry*, *8*(April). https://doi.org/10.9790/264X-08024449

Feng, X., Ameer, K., Ramachandraiah, K., Wu, Z., Huo, N., Bai, X., Nie, W., & Jiang, G. (2022). Effects of papaya (Carica papaya L.) seed supplementation on quality attributes, adsorption capacities, and in vitro starch digestibility of wheat bread. *Journal of Food Measurement and Characterization*, *16*(4), 3226-3239. https://doi.org/10.1007/s11694-022-01415-0

Gonçalves Rodrigues, L. G., Mazzutti, S., Vitali, L., Micke, G. A., & Ferreira, S. R. S. (2019). Recovery of bioactive phenolic compounds from papaya seeds agroindustrial residue using subcritical water extraction. *Biocatalysis and Agricultural Biotechnology*, *22*, 101367. https://doi.org/10.1016/j.bcab.2019.101367

Gonza, T., Cha, P., & Rodrı, I. (2011). Antifungal Activity in Ethanolic Extracts of Carica papaya L . cv . Maradol Leaves and Seeds. *Indian J Microbiol*, *51*(1), 54-60. https://doi.org/10.1007/s12088-011-0086-5

Gunde, M., & Amnerkar, N. (2016). Nutritional , medicinal and pharmacological properties of papaya ( Carica papaya linn. ) : A review. *Journal of Innovations in Pharmaceuticals and Biological Sciences*, *3*(1), 162-169. https://jipbs.com/index.php/journal/article/view/124

Haber, R. A., Garcia, R. D., Hernandez, J. N., Jamieson, S., Mondal, A., & Bishayee, A. (2022). Papaya ( Carica papaya L .) for cancer prevention : Progress and promise. *Critical Reviews in Food Science and Nutrition*, *0*(0), 1-21. https://doi.org/10.1080/10408398.2022.2079607

Kanupriya, J., M, S., Shivashankara, K. S., & Vasugi, C. (2016). Biochemical properties of yellow and red pulped papaya and its validation by molecular markers. *Indian Journal of Horticulture*, *73*(3), 315-318. https://doi.org/10.5958/0974-0112.2016.00070.0

Khaw, K. Y., Shaw, P. N., Parat, M. O., Pandey, S., & Falconer, J. R. (2020). Compound identification and in vitro cytotoxicity of the supercritical carbon dioxide extract of papaya freeze-dried leaf juice. *Processes*, *8*(5). https://doi.org/10.3390/PR8050610

Kong, Y. R., Jong, Y. X., Balakrishnan, M., Bok, Z. K., Weng, J. K. K., Tay, K. C., Goh, B. H., Ong, Y. S., Chan, K. G., Lee, L. H., & Khaw, K. Y. (2021). Beneficial role of carica papaya extracts and phytochemicals on oxidative stress and related diseases: A mini review. *Biology*, *10*(4), 1-20. https://doi.org/10.3390/biology10040287

Kouao, T. A., Kouame, B. A., Ouattara, Z. A., Mamyrbekova-Bekro, J. A., Bighelli, A., Tomi, F., & Bekro, Y.-A. (2021). Chemical characterisation of essential oils of leaves of two Solanaceae: Solanum rugosum and Solanum erianthum from Côte d’Ivoire. *Natural Product Research*, *35*(14), 2420-2423. https://doi.org/10.1080/14786419.2019.1672064

Kumar, M., Saurabh, V., Tomar, M., Hasan, M., Changan, S., Sasi, M., Maheshwari, C., Prajapati, U., Singh, S., Prajapat, R. K., Dhumal, S., Punia, S., Amarowicz, R., & Mekhemar, M. (2021). Mango (Mangifera indica L.) Leaves: Nutritional Composition, Phytochemical Profile, and Health-Promoting Bioactivities. *Antioxidants (Basel)*, *10*(2). https://doi.org/10.3390/antiox10020299

Kumaree, K. K., Anthikapalli, N. V. A., & Prasansuklab, A. (2023). In silico screening for potential inhibitors from the phytocompounds of Carica papaya against Zika virus NS5 protein. *F1000Research*, *12*, 655. https://doi.org/10.12688/f1000research.134956.1

Lara-Abia, S., Lobo-Rodrigo, G., Welti-Chanes, J., & Pilar Cano, M. (2021). Carotenoid and carotenoid ester profile and their deposition in plastids in fruits of new papaya (Carica papaya l.) varieties from the canary islands. *Foods*, *10*(2), 1-26. https://doi.org/10.3390/foods10020434

Mehmood, H., Mehmood, J., & Zulfiqar, N. (2024). Exploring the phytochemistry and pharmacology of Mangifera indica L. (Mango) leaves: A review. *International Journal of Plant Based Pharmaceuticals*, *4*(1), 9-18. https://doi.org/10.29228/ijpbp.38

Muntholib, Sulistyaningrum, D., Subandi, & Marfu’ah, S. (2020). Identification of flavonoid isolates of papaya (Carica papaya L.) seed and their activity as pancreatic lipase inhibitors. *AIP Conference Proceedings*, *2231*(1). https://doi.org/10.1063/5.0003456

Ntakoulas, D. D., Pasias, I. N., Raptopoulou, K. G., Dimitriou, G., & Proestos, C. (2023). Phytochemical screening of Psidium guajava and Carica papaya leaves aqueous extracts cultivated in Greece and their potential as health boosters. In *Exploration of Foods and Foodomics* (pp. 5-14). https://doi.org/10.37349/eff.2023.00002

Pambhar, V., Navgeet, M., Mehta, A., Mathur, M., Kumawat, D. C., Mangalia, R., Verma, A., & Patyal, A. (2022). Effect of doxycycline and doxycycline with Carica papaya on thrombocytopenia and leucopenia in acute dengue fever patients. *Journal of Family Medicine and Primary Care*. https://doi.org/10.4103/jfmpc.jfmpc

Pertiwi, D., Hafiz, I., & Salma, R. (2019). Antibacterial Activity of Ethanol Extract of Papaya leaves (Carica papaya L.) Gel against P.acnes. *Indonesian Journal of Pharmaceutical and Clinical Research (IDJPCR*, *2*(1), 1-6. https://doi.org/https://doi.org/10.32734/idjpcr.v2i1.869

Quintana, S. E., Salas, S., & García-Zapateiro, L. A. (2021). Bioactive compounds of mango (Mangifera indica): a review of extraction technologies and chemical constituents. *Journal of the Science of Food and Agriculture*, *101*(15), 6186-6192. https://doi.org/10.1002/jsfa.11455

Sani, M. S. A., Bakar, J., Rahman, R. A., & Abas, F. (2020). Effects of Coated Capillary Column, Derivatization, and Temperature Programming on the Identification of Carica papaya Seed Extract Composition Using GC/MS Analysis. *Journal of Analysis and Testing*, *4*(1), 23-34. https://doi.org/10.1007/s41664-020-00118-z

Soorya, R., Dhamodaran, P., Rajesh, R., & Duraisamy, B. (2017). In Silico Studies of The Secondary Metabolites of Solanum Torvum Sw. for Their Antiasthmatic Activity. *International Journal of Current Pharmaceutical Research*, *9*, 38. https://doi.org/10.22159/ijcpr.2017v9i4.20759

Trébissou, J. N. D., Bla, B., Yapo francis, A., Yapi, H. F., & Djaman, J. (2014). Therapeutic survey on traditional treatment of Buruli ulcer in Côte d'Ivoire. *J. Microbiol. Biotech. Res*.*,* 4, 52-56.

Vaithilingam, S., Vivekanandan, L., & Krishna, M. (2021). In Silico Study of Pubchem Compounds for Solanum torvum as Antiviral Agent against SARS-CoV-2. *The Open COVID Journal*, *1*, 235-242. https://doi.org/10.2174/2666958702101010235

Zhang, W., Pan, X., Fu, J., Cheng, W., & Lin, H. (2024). Phytochemicals derived from Nicotiana tabacum L . plant contribute to pharmaceutical development. *Frontiers in Pharmacology*, *April*, 1-19. https://doi.org/10.3389/fphar.2024.1372456

Zou, X., Bk, A., Abu-Izneid, T., Aziz, A., Devnath, P., Rauf, A., Mitra, S., Emran, T. B., Mujawah, A. A. H., Lorenzo, J. M., Mubarak, M. S., Wilairatana, P., & Suleria, H. A. R. (2021). Current advances of functional phytochemicals in Nicotiana plant and related potential value of tobacco processing waste: A review. *Biomedicine and Pharmacotherapy*, *143*(August). https://doi.org/10.1016/j.biopha.2021.112191
